# Regulation of Hematopoietic Stem Cell Behavior by the Nanostructured Presentation of Extracellular Matrix Components

**DOI:** 10.1371/journal.pone.0054778

**Published:** 2013-02-06

**Authors:** Christine Anna Muth, Carolin Steinl, Gerd Klein, Cornelia Lee-Thedieck

**Affiliations:** 1 Department of New Materials and Biosystems, Max Planck Institute for Intelligent Systems, Stuttgart, Germany; 2 Department of Biophysical Chemistry, University of Heidelberg, Heidelberg, Germany; 3 Section for Transplantation Immunology and Immunohematology, Center for Medical Research, University of Tübingen, Tübingen, Germany; 4 Institute of Functional Interfaces, Karlsruhe Institute of Technology (KIT), Eggenstein-Leopoldshafen, Germany; University of Frankfurt - University Hospital Frankfurt, Germany

## Abstract

Hematopoietic stem cells (HSCs) are maintained in stem cell niches, which regulate stem cell fate. Extracellular matrix (ECM) molecules, which are an essential part of these niches, can actively modulate cell functions. However, only little is known on the impact of ECM ligands on HSCs in a biomimetic environment defined on the nanometer-scale level. Here, we show that human hematopoietic stem and progenitor cell (HSPC) adhesion depends on the type of ligand, i.e., the type of ECM molecule, and the lateral, nanometer-scaled distance between the ligands (while the ligand type influenced the dependency on the latter). For small fibronectin (FN)–derived peptide ligands such as RGD and LDV the critical adhesive interligand distance for HSPCs was below 45 nm. FN-derived (FN type III 7–10) and osteopontin-derived protein domains also supported cell adhesion at greater distances. We found that the expression of the ECM protein thrombospondin-2 (THBS2) in HSPCs depends on the presence of the ligand type and its nanostructured presentation. Functionally, THBS2 proved to mediate adhesion of HSPCs. In conclusion, the present study shows that HSPCs are sensitive to the nanostructure of their microenvironment and that they are able to actively modulate their environment by secreting ECM factors.

## Introduction

Hematopoietic stem cells (HSCs) are located in specific environments in the bone marrow, i.e. the stem cell niches. Specialized niche cells, extracellular matrix (ECM) and soluble factors play essential roles in regulating HSC function and maintenance. However, to which extent those factors contribute to the functionality of the bone marrow stem cell niches and how they are regulated remains uncertain [1].

Evidence has been found that in addition to the niche regulating stem cell behavior, stem cells and their progeny also actively modulate their niche [2], [3]. Several HSC niches have been described in mice: (i) the endosteal niche containing osteoblastic cells as the major HSC-supporting cell type [4], [5], [6], (ii) the perivascular niche, in which HSCs are influenced by vascular and perivascular cells [7], [8], [9], and (iii) a niche formed by nestin^+^ mesenchymal stem cells [10]. In all niches mesenchymal stem cells play an important role [11]. To date, the relevance of each of these niches for individual HSC functions is under debate.

The ECM is of particular interest, because it can induce diverse cell responses [12]. The bone marrow ECM is a complex composition of collagens, proteoglycans, glycosaminoglycans, and glycoproteins such as fibronectin (FN), osteopontin (OPN), laminins and thrombospondins (THBS) [1], [13]. Different ECM molecules can influence adhesion, proliferation, survival, migration and differentiation of stem cells [14]. In elegant studies the Werner group has shown in a biomimetic setup that HSC fate decisions depend on the delicate balance of adhesive interactions with ECM components and stimulation by soluble factors [15], [16], [17]. Among the studied ECM-derived ligands FN proved to mediate the strongest adhesion of HSCs to material surfaces [15].

Many ECM proteins mediate cell adhesion via integrin receptors [18]. Integrins are heterodimers containing two distinct subunits, referred to as the α and β chain. So far, 18 α and 8 β chains have been described. Twenty-four unique receptors types, generated through different subunit combinations, are known [18], [19]. Integrins can be categorized into different subsets according to which α or β chain they contain and the class of ECM proteins to which they bind [20]. It has been shown that the integrin β1 chain is essential for hematopoietic stem and progenitor cell (HSPC) homing and migration [21], [22], and that blocking the integrin α_5_ chain inhibits HSPC adhesion to FN [15]. Integrin α_4_β_1_- and α_5_β_1_-mediated binding to FN has also been described to impact HSC growth [23], [24].

FN is a well-characterized ECM protein, which is present in the bone marrow. Because FN has been described to promote as well as to inhibit HSC proliferation [17], [25], [26], [27], its significance for HSC function remains controversial. One possible explanation for these contradictory findings may be the existence of different conformations of the FN molecules, depending on the type of surface they are immobilized on [28]. The FN molecule is composed of three types of modules (type I, II, III). Short peptide motifs within these modules have been identified as key elements of the integrin receptor recognition sites of FN. The 10^th^ type III module contains an RGD sequence (Fig. S1A). This motif is the best-studied minimal cell adhesive sequence [29]. It is situated in a flexible loop structure between two strands, in close proximity to the synergy sequence PHSRN of the 9^th^ type III module [30]. This synergy sequence enhances the interaction stability of α_5_β_1_ integrin with FN [31]. In addition, the alternatively spliced V region in the C-terminal cell binding domain of FN contains an LDV motif that is recognized by hematopoietic cells via their α_4_β_1_ integrin receptor [32]. Figure S1 shows a schematic cartoon of FN and FN-derived ligands that were applied in this study, including the location of the cell-binding domains.

Another important ECM molecule of the hematopoietic microenvironment is OPN, which has been described as a negative regulator of the murine HSC pool size [33], [34]. The N-terminal thrombin fragment of OPN (amino acids 17–168) contains an RGD sequence and thrombin-cleaved OPN can regulate HSPC functions (e.g., migration and homing) through interactions with α_9_β_1_ and α_4_β_1_ integrins [35].

The geometric arrangement of ligands on the nanometer scale and the matrix elasticity are as important for HSC function as the composition of the ECM [36], [37], [38], [39]. It is well known that the nanopatterned spatial presentation of ECM ligands influences adhesion, migration and focal adhesion assembly of fully differentiated tissue cells such as fibroblasts and osteoblasts [40], [41]. However, only little is known about the impact of ECM ligands on HSCs in a biomimetic environment defined at the nanometer-scale level. It has been shown that synthetic nanostructured environments influence HSC adhesion, lipid raft clustering and expansion [36], [42]. The aim of the current study was to identify ECM signals that guide HSC function in the context of a nanostructured environment. In order to mimic the natural microenvironment of cells, which is structured from the micro- to the nanometer-scale, biocompatible materials allowing the control over ligand choice, ligand orientation and receptor clustering in the nanometer range are essential [43], [44]. These requirements were fulfilled in the current study by using hydrogel-supported gold nanopatterns equipped with bioactive molecules. Quasi-hexagonally ordered gold nanoparticle (NP) arrays were produced using block copolymer micelle nanolithography (BCML). The distances between the NPs were adjusted to values between 20 and 110 nm by varying the production parameters [45]. NP diameters ranged from 6 to 8 nm. The gold NP arrays were embedded in polyethylene glycol (PEG) hydrogels [46]. PEG is a nontoxic, biologically inert and protein-repellent material, making it highly useful for biological applications [47], [48]. Since a single gold NP is smaller than a singular integrin receptor (∼ 10 nm in diameter [49]), theoretically only one receptor should be able to bind per biofunctionalized NP due to steric hindrance. Thus, using this nanopatterned PEG hydrogel system, an one-to-one interaction between a single receptor and a single biofunctionalized gold NP is possible and lateral receptor clustering can be controlled [50].

In order to unravel the significance of matrix nanostructure for the HSC niche function, we investigated the influence of different ECM ligands and nanopatterns on HSPC adhesion, proliferation, differentiation and gene expression.

## Results

### Characterization of Biofunctionalized, Nanopatterned Hydrogels

**Figure 1:**
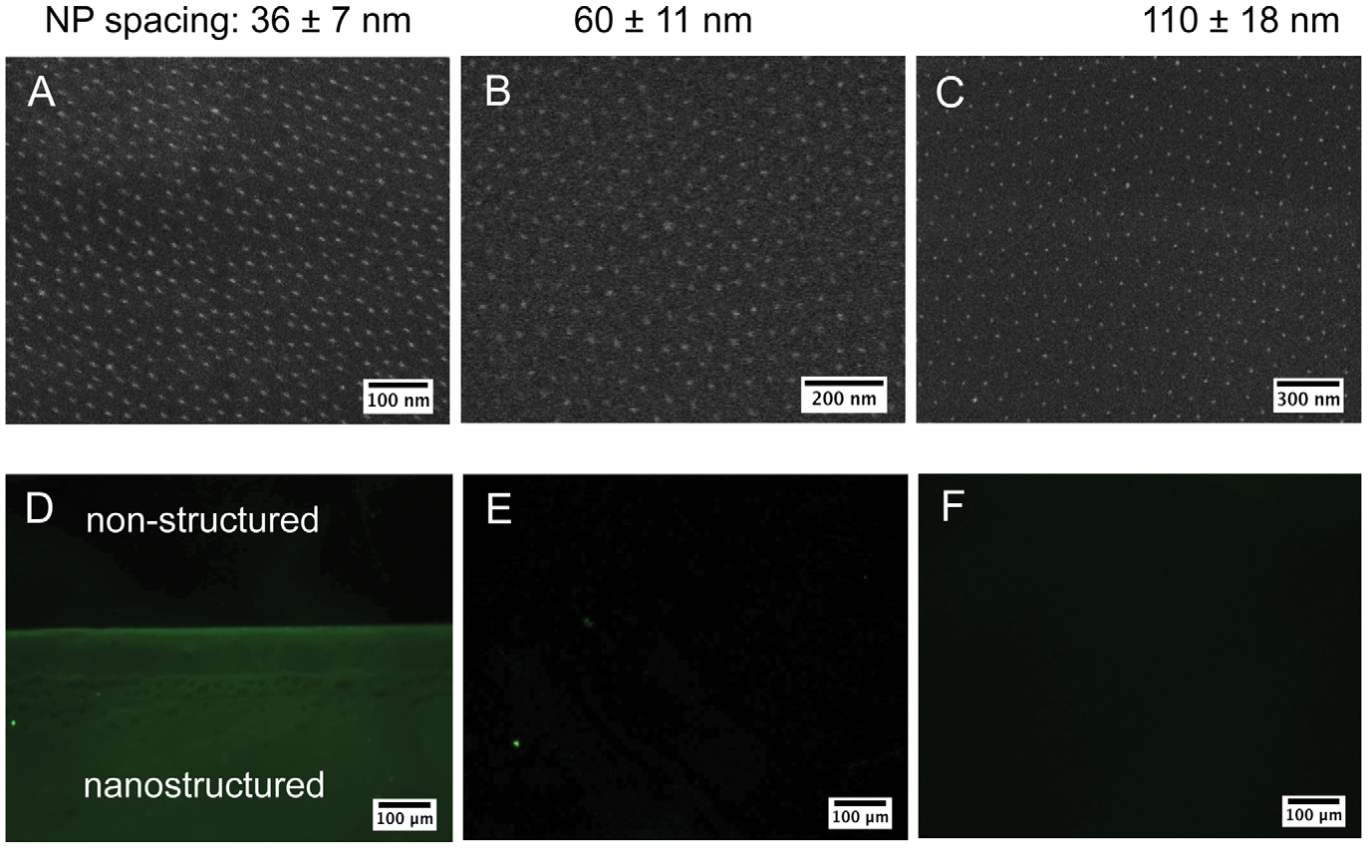
Nanopatterned and biofunctionalized PEG hydrogels. (A–C) Cryo-SEM images of the quasi-hexagonally ordered gold NP patterns on PEG hydrogels with interparticle distances of (A) 36±7 nm, (B) 60±11 nm and (C) 110±18 nm. (D–F) Micrographs of fluorescently labeled, FNRGD-functionalized nanostructured hydrogels. Images of the border between the nanostructured area in the lower part of the micrographs and the unstructured area visible in the upper part are shown. (D) The FNRGD domain on biofunctionalized hydrogels was detected with the help of specific primary antibodies. Controls were produced by (E) substituting nickel with EDTA during functionalization and by (F) omitting the primary antibody during the staining procedure. One representative experiment out of 3 is shown.

Gold nanopatterned PEG hydrogels were produced and biofunctionalized with ligands that are present in the bone marrow ECM to mimic properties of the ECM environment. The basis for ligand presentation was set by gold NPs, which were evenly spread in a predefined pattern with a lateral particle distance between 36±7 and 110±18 nm (Fig. 1A–C). N-terminally His-tagged protein domains were bound to the gold NPs via a thiolated NTA-linker in a site-specific and oriented manner [51]. The applied protein domains were derived from FN or OPN. The OPN domain, called OPNs, corresponds to the thrombin-cleaved OPN fragment (amino acids 17–168) containing a C-terminal RGD sequence. The FN domain, called FNRGD, consists of the type III modules 7–10 and contains the characteristic integrin-binding motif RGD. This protein domain was used in a functionally intact version (FNRGD) equipped with the RGD sequence and in a mutated version (FNΔRGD) lacking this crucial integrin-binding motif. Fluorescent labeling of nanostructured hydrogels functionalized with FNRGD showed the immobilization of the domains to be selective for the nanostructured part of the hydrogel, not for the unstructured internal control (Fig. 1D). This suggests a specific functionalization of the gold NPs and not of the PEG hydrogel itself. As expected, when using EDTA instead of Ni^2+^ during coupling, binding did not occur and no fluorescence above background noise could be detected (Fig. 1E). This indicates His-tag-mediated oriented and site-specific binding. Controls where the primary antibody was omitted showed no unspecific antibody binding (Fig. 1F).

### Contacts of Hematopoietic Cells to Biofuntionalized Gold NPs

**Figure 2:**
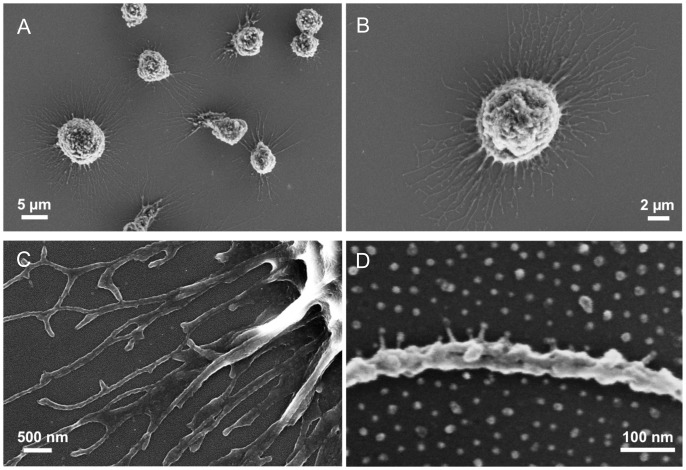
Cell morphology on nanostructured glass substrates. SEM images of critical point dried KG-1a cells on nanostructured, PEG-passivated, cRGD-functionalized substrates with interparticle distances of 36±7 nm. Magnification increases from A to D.

Nanostructured PEG hydrogels were functionalized with a cyclic RGD peptide (cRGD) featuring the RGD sequence that is usually located in a loop region of FN (Fig. S1). KG-1a cells were allowed to adhere to these substrates for 1 h. Scanning electron microscopy (SEM) revealed that KG-1a cells kept their round morphology upon adhesion and were in close contact with the cRGD-functionalized nanostructured substrates through their filopodia (Fig. S2). Higher magnification imaging of cells on hydrogel substrates with sufficient resolution to monitor cellular structures and the substrate nanostructure at the same time was impossible due to electrical charging of the samples during SEM measurements. To overcome this limitation BCML-nanostructured glass substrates were employed. The glass background of these substrates was passivated with PEG in order to prevent non-specific cell adhesion and protein absorption and the gold NPs were functionalized with cRGD similar to the hydrogels. The morphology of cells imaged on glass and hydrogels appeared to be identical in SEM (Fig. 2A, B and S2). Higher magnification of the filopodia showed that the cells were in contact with the biofunctionalized gold NPs and not with the PEG-passivated area in between the NPs (Fig. 2C, D). These results indicate that hematopoietic cells are able to sense nanostructures on cRGD-functionalized surfaces.

### Impact of ECM Ligand Type and Spacing on KG-1a and HSPC Adhesion

**Figure 3:**
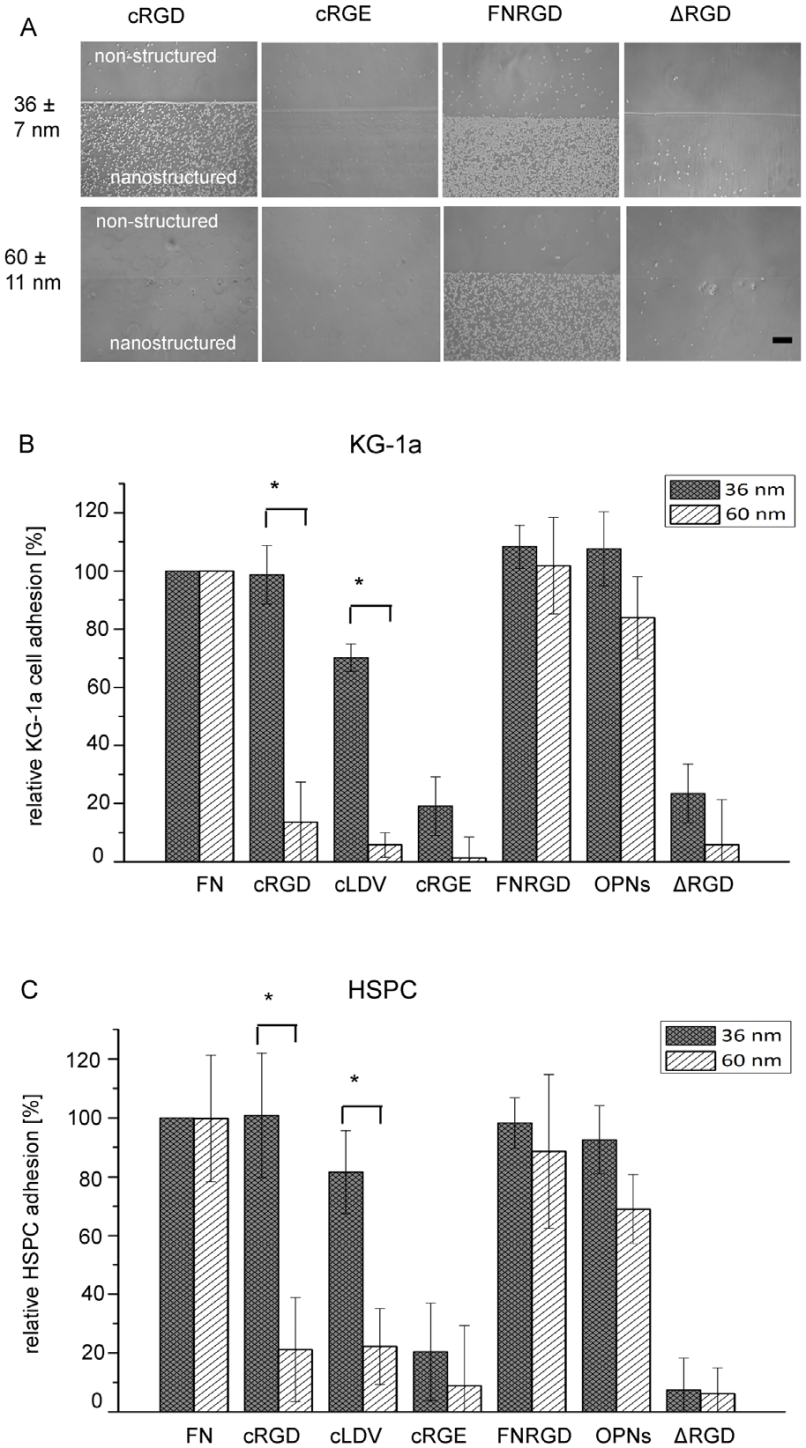
Adhesion of KG-1a cells and HSPC to nanopatterned, biofunctionalized PEG hydrogels. Cells were plated on hydrogels in adhesion media and evaluated by phase contrast microscopy or Cy Quant cell quantification after 1 h of incubation. (A) Microscopic images of KG-1a cells on biofunctionalized PEG hydrogels. Cells appear as bright spots on a gray background. The area in the lower part of each image is nanostructured and the upper area is unstructured (internal control). The name of the applied ligand is given above each image. Cell adhesion to NP arrays with a distance of 36±7 nm or 60±11 nm are shown in the upper and lower row of images, respectively. FNΔRGD is abbreviated with “ΔRGD”. One representative experiment out of 4 is shown. Scale bar = 200 µm. (B) Relative quantification of KG-1a cell adhesion and (C) HSPC adhesion to different ligands on NP arrays with 36±7 nm (filled columns) or 60±11 nm (diagonally striped columns) spacing. On the y-axis the number of adherent cells normalized to the value for adhesion to full-length FN is plotted. The different ligands are indicated on the x-axis. N_independent experiments_ = 4, each experiment carried out in technical duplicates; error bars = standard deviation of the mean; * = significant p value <0.05 in Wilcoxon rank sum test.

The combined influence of nanostructural and biochemical parameters on cell adhesion *in vitro* was studied on nanopatterned, biofunctionalized PEG hydrogels. The nanostructured hydrogels were biofunctionalized with cRGD, cLDV, FNRGD, OPNs or the non-functional control ligands (cRGE, FNΔRGD). Micrographs of KG-1a cells on representative cRGD-, cRGE-, FNRGD- or FNΔRGD-functionalized nanostructured hydrogels are shown in Fig. 3A. In general, KG-1a cells exclusively adhered to the nanostructured, rather than the unstructured, area of the hydrogels. Furthermore, adhesion was only observed to the functional ligands cRGD and FNRGD, while adhesion to the control ligands (cRGE, FNΔRGD) was negligible to non-existent (Fig. 3A upper panel). KG-1a cells adhered to surfaces with cRGD and FNRGD at 36±7 nm interparticle spacing. On surfaces with 60±11 nm interparticle spacing KG-1a cells were unable to adhere to cRGD, while adhesion to the FNRGD domain was similar to that on the surfaces with an interparticle spacing of 36±7 nm (Fig. 3A lower panel).

To compare hematopoietic cell adhesion to nanostructured surfaces varying in interparticle distance and ligand type an assay based on the accurate determination of DNA content was applied. The number of adhering cells was normalized to the number of cells adhering to full-length FN (defined as 100% adhesion). On hydrogels functionalized with cRGD, FNRGD or OPNs at 36±7 nm NP spacing, the number of adhering HSPC and KG-1a cells was comparable to the number adhering to a surface functionalized with the full-length FN protein (100%). On hydrogels functionalized with cLDV cell adhesion was generally lower compared to that of the other adhesive ligands (Fig. 3B, C). Only 1–22% adhesion could be observed on surfaces with peptide ligands spaced apart 60±11 nm in comparison to full-length FN. This value is comparable to that observed for the non-adhesive control ligands. In contrast, cells were able to successfully adhere to hydrogels biofunctionalized with protein domains at 60±11 nm spacing.

In summary, hematopoietic cells were able to bind to all investigated adhesive ligands when these were located close enough to each other (36±7 nm). At 60±11 nm interparticle spacing only the protein domains elicited substantial cell adhesion. The critical interparticle distance (i.e., the maximal distance), which was still able to support KG1a cell adhesion, was determined to be between 37±7 nm and 45±9 nm for cRGD (Fig. S3), between 85±14 nm and 110±18 nm for the FNRGD domain (Fig. S4A) and between 75±13 nm and 85±14 nm for the OPNs domain (Fig. S4B). Based on these results, for the following experiments we focused on the ligands mediating the highest cell adhesion, which were cRGD, the FN domains and OPNs.

### Integrin Mediated HSPC Adhesion to FN

Eleven different integrins with the ability to bind to FN have been described [52]. Eight of these bind to the 9^th^ and 10^th^ type III modules, which were contained in the FN domains we used. To investigate which integrins are responsible for HSPC binding to surfaces biofunctionalized with FN we used RGD to block all RGD-binding integrins, specific antibodies to block particular integrin chains (anti-β_1_, anti-α_4_ and anti-α_5_) and an isotype control without specificity for the target cells.

**Figure 4:**
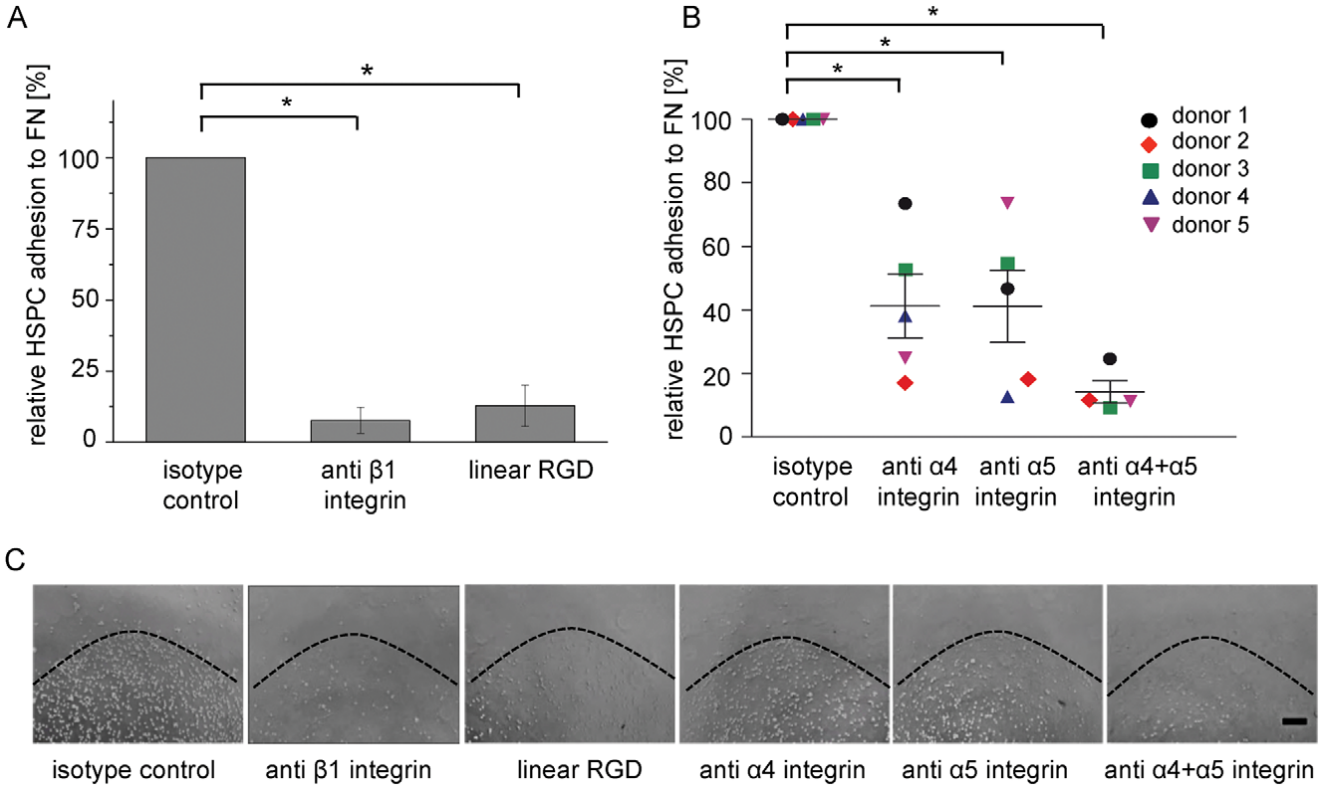
Integrin-mediated HSPC adhesion to FN. HSPCs were preincubated with integrin-specific antibodies or a linear RGD peptide for 1 h and plated onto an adsorbed FN spot. After nonadherent cells were removed, the spots were imaged and the adherent cells counted. (A) Relative HSPC adhesion to FN was either inhibited by preincubation with antibodies blocking the β1 integrin chain or with a linear RGD peptide. The isotype control (set to 100%) shows cell adhesion to FN after preincubation with isotype control antibodies. N_independent experiments = _4. (B) HSPC adhesion to FN was significantly reduced when inhibiting either the α_4_ integrin chain, the α_5_ integrin chain or both. N_independent experiments = _5. (A, B) Error bars = standard error of the mean; * = significant p value <0.05 in Wilcoxon rank sum test. (C) Representative microscopic images of HSPC adhesion to FN spots. Cells are visible as white spots in the lower part of each image, the dashed black line indicates the border of the FN spot. Scale bar = 200 µm.

The addition of soluble RGD prevented HSPC adhesion, confirming that HSPCs bind to the RGD motif in FN. Furthermore the addition of function-blocking anti-β_1_ integrin antibodies also prevented HSPC adhesion to FN (Fig. 4A, C). Three of the RGD-binding integrins (α_5_β_1_, α_V_β_1_ and α_8_β_1_) contain the β_1_ chain [18], [52], but only integrin α_5_β_1_ is known to be expressed by HSPCs. This suggests that HSPCs bind to the RGD sequence in FN via the integrin α_5_β_1_ receptor. Interestingly, the effect of α_5_ integrin chain inhibition was weaker than β_1_ integrin chain inhibition (Fig. 4B, C), indicating that additional β_1_ integrins besides α_5_β_1_ are involved in HSPC adhesion to FN. HSPCs also express α_4_β_1_ integrin, which binds to the carboxy-terminal cell binding domain (containing the LDV motif) of FN [32]. The effect of the combined inhibition of α_4_ and α_5_ integrin chains was similar to the result of inhibiting only the β_1_ chain, leading to similarly weak cell adhesion to FN. The contribution of each of the two α integrins to HSPC adhesion was donor-dependent (Fig. 4B). As observed for full-length FN, adhesion to the FNRGD domain could be blocked by the addition of β_1_ integrin antibodies (Fig. S5).

### Effect of FN-derived and OPN-derived Ligands on HSPC Proliferation and Differentiation

To investigate the influence of ligands and nanostructure on HSPC fate, colony forming assays were performed on adhesive cRGD- and non-adhesive cRGE-functionalized nanostructured PEG hydrogels. No effect of the ligand on the number or type of the formed colonies could be observed (Fig. S6). In order to identify ligands, which are potent in influencing HSPC proliferation and differentiation, we performed CFSE-proliferation experiments and differentiation analyses with continuous layers of ligands. In this setting the ligand density is much higher than on nanopatterned substrates and the resulting surfaces are better comparable to the ones used in other studies investigating the impact of ECM ligands on HSPCs [15], [25], [26], [27], [34].

Continuous gold surfaces were biofunctionalized with cRGD, cRGE, FNRGD, FNΔRGD or OPNs. Cell proliferation on these surfaces was compared to cells growing on unfunctionalized gold surfaces. Freshly isolated HSPCs were labeled with CFSE and incubated on the biofunctionalized gold surfaces. After 4 and 7 days of pre-culture on the different surfaces the amount of retained CFSE and CD34 protein expression were determined by flow cytometry. All 5 biofunctionalized surfaces gave similar results and were comparable to the gold control surface with regard to cell expansion (Fig. S7A, C) and CD34 expression (Fig. S7B, D).

**Figure 5:**
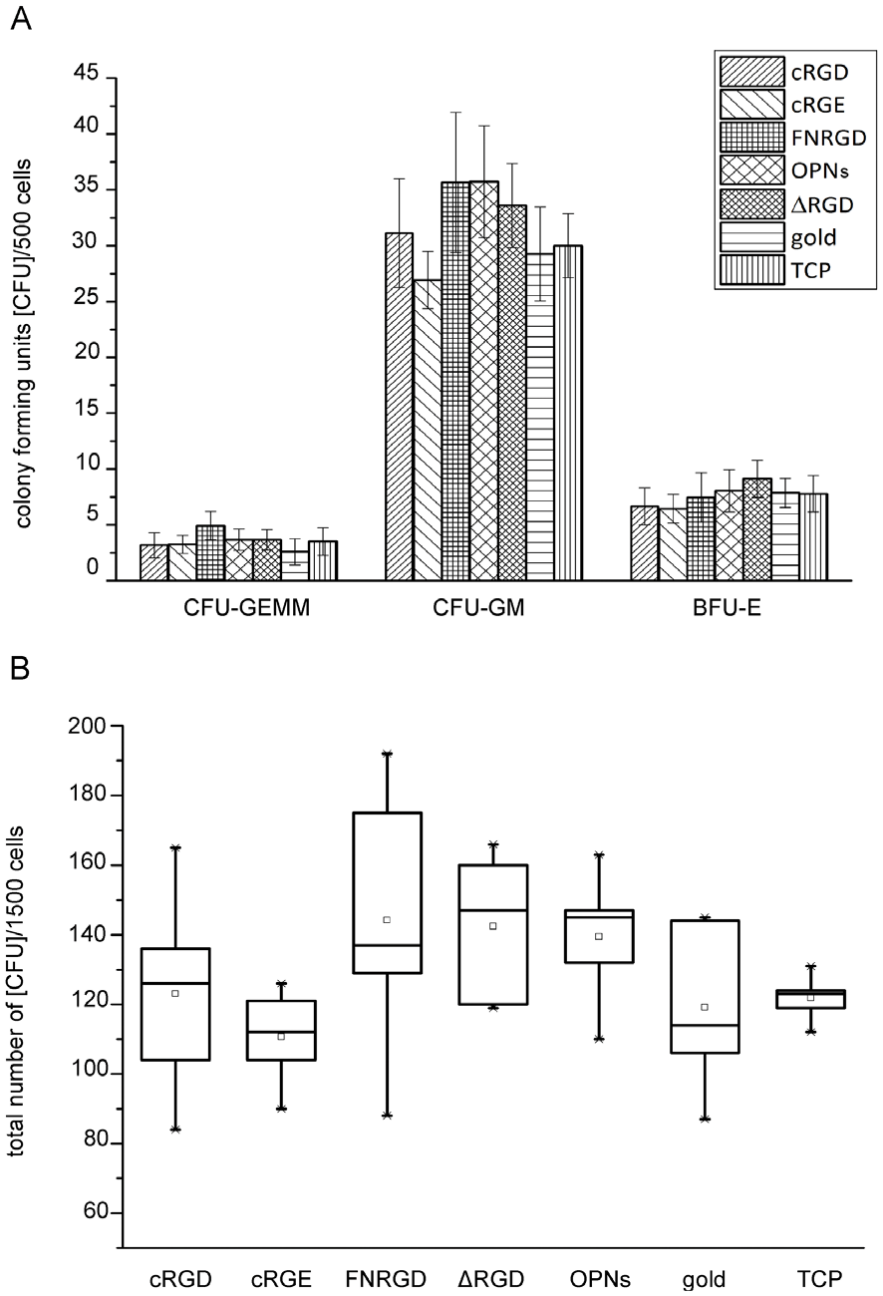
Determination of HSPC differentiation in colony forming assays. (A) Colony forming units of precultured HSPCs on glass slides biofunctionalized with different ligands. After 7–10 days preculture, colony forming assays were performed in triplicates. Colonies were distinguished into CFU-GEMM (*colony forming unit granulocyte, erythroid, macrophage, megakaryocyte*), CFU-GM (*colony forming unit granulocyte, macrophage*) and BFU-E (*burst forming unit erythroid*) after 2 weeks. N_independent experiments = _5 in technical triplicates; error bars = standard error of the mean. (B) Box Plot of the total number of colony forming units formed by 1500 precultured HSPCs on glass slides biofunctionalized with different ligands. N_independent experiments = _5; TCP = tissue culture plastic, gold = continuous gold film on glass, FNΔRGD is abbreviated with “ΔRGD”.

Colony forming assays were performed after 7 to 10 days of pre-cultivation on the different surfaces to test the influence of the ligands on HSPC differentiation. No significant differences in the number or type of colonies were found (Fig. 5), indicating that the immobilized ECM-derived ligands had no impact on HSPC differentiation.

### Effects of Ligand Type and Nanostructure on the Expression of THBS2

The investigated ligands and their nano-scale spacing influenced integrin-mediated cell adhesion. Quantitative RT-PCR screening for the expression of integrin ligands revealed thrombospondin-2 (THBS2) as a gene that is regulated in its expression by the FN-derived ligands and the lateral interligand distance.

**Figure 6:**
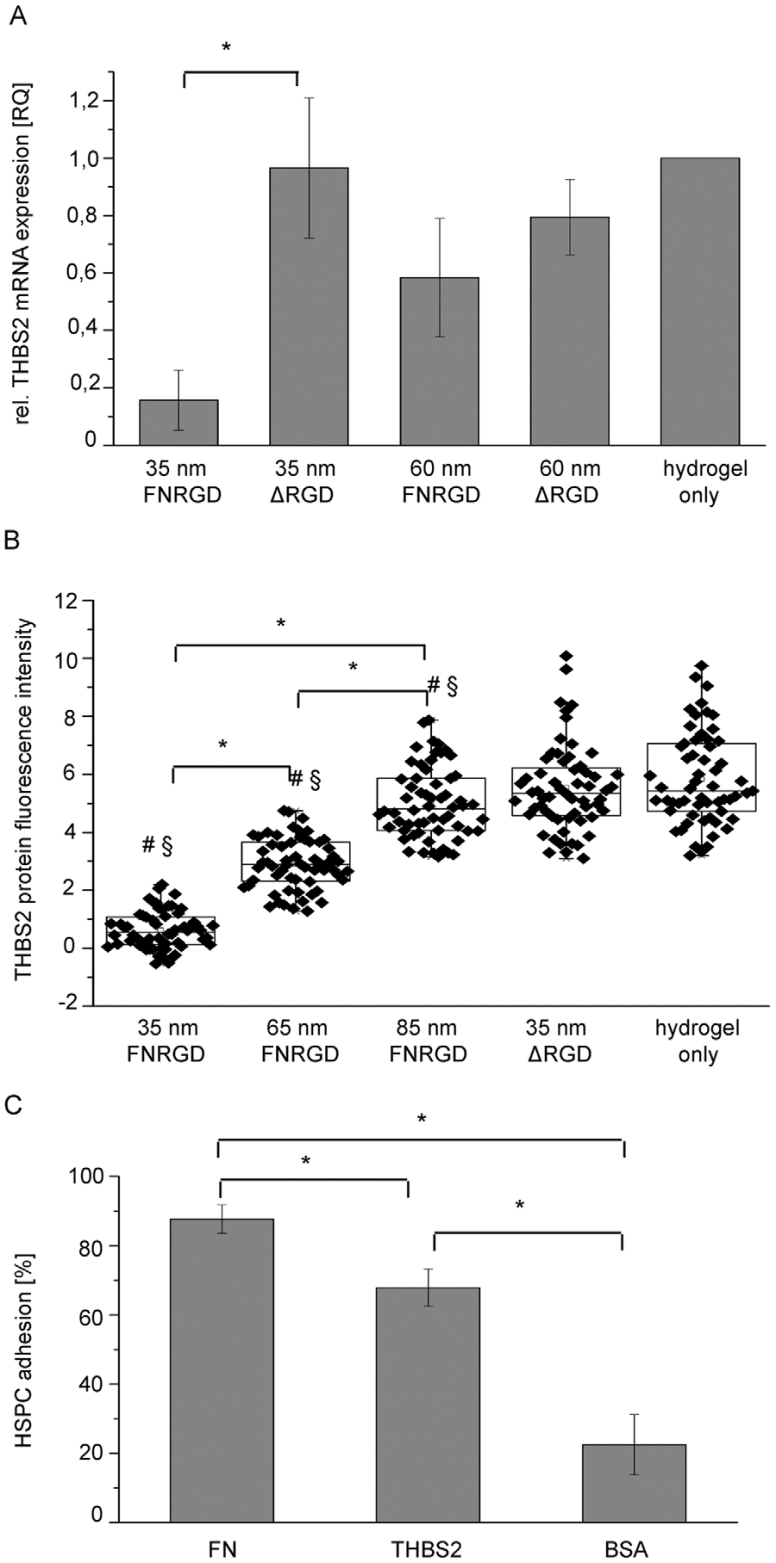
Influence of nanostructured matrices on THBS2 expression by HSPC. (A) Relative gene expression of THBS2 in HSPC incubated for 140 min on nanostructured PEG hydrogels biofunctionalized with FNRGD or FNΔRGD (abbreviated with “ΔRGD”). RQ values were normalized to the unstructured hydrogel controls and are plotted on the y-axis. The different functionalized and nanostructured substrates are indicated on the x-axis. N_independent experiments_ = 4; error bars = standard error of the mean; * = significant p value <0.05 in Wilcoxon rank sum test. (B) Immunofluorescence staining of THBS2 protein in HSPCs incubated for 13 h on nanostructured, biofunctionalized PEG hydrogels. Fluorescence intensity was measured per cell by applying Image J software and is plotted on the y-axis. N_cells_ = 60 from 3 donors (3×20); data are presented as box plots overlaid with individual data points (black squares/diamonds); * = significant p value <0.001; # = significant p value <0.001 to cells on hydrogel only; § = significant p value <0.001 to cells on 35±7 nm FNΔRGD (abbreviated with “ΔRGD”) biofunctionalized hydrogels. Two-tailed, unpaired Student’s t-test. (C) HSPC adhesion to full-length FN, BSA or recombinant THBS2 protein in percent of the total applied cell number per well. N_independent experiments_ = 5, each experiment carried out in technical triplicates; error bars = standard deviation of the mean; * = significant p value <0.01 in Wilcoxon rank sum test.

HSPCs expressed THBS2 mRNA on blank, ligandless PEG hydrogels, which were unable to elicit integrin signaling (relative quantification value (RQ) set to 1, Fig. 6A). THBS2 expression was similar (RQ = 0.97) on FNΔRGD functionalized hydrogels with interparticle spacings of 35±7 nm, but significantly lower (RQ = 0.16) on 35±7 nm hydrogels functionalized with FNRGD. On 60±11 nm FNRGD-functionalized hydrogels THBS2 expression was at an RQ of 0.58. This value lies between the relative expression on 35±7 nm hydrogels functionalized with FNRGD and those functionalized with FNΔRGD, but is not significantly different to either value. This indicates that the lateral spacing of FNRGD and the presence or absence of integrin activation regulates THBS2 mRNA expression.

In a next step, these findings were verified on the protein level by immunofluorescence staining of THBS2 in HSPCs that were incubated on nanostructured hydrogels. THBS2 protein expression on nanostructured hydrogels was regulated similar to the mRNA expression (Fig. 6B, Fig. S8). The highest protein expression was found on blank or FNΔRGD functionalized hydrogels. On 35±7 nm nanostructured FNRGD-functionalized hydrogels none or only minor THBS2 expression could be observed. Significantly more THBS2 protein expression was detected on FNRGD-functionalized hydrogels with interparticle distances of 65±11 nm, and even more at 85±14 nm. On these matrices the lateral distance between the FNRGD-functionalized particles was increased, resulting in a higher distance between the targeted cellular integrins. This shows that THBS2 protein expression was enhanced in the absence of the RGD sequence in FN and by increasing the lateral distance between integrins.

To reveal a possible function of THBS2 in the context of the HSC microenvironment, adhesion assays comparing cell surfaces bearing THBS2, FN or BSA were performed. HSPC adhesion was significantly higher on THBS2 (68% of all applied cells bound) and FN (88%) functionalized surfaces than on control surfaces with BSA (22%) (Fig. 6C). This indicates that THBS2 functions as an adhesion-mediating molecule for HSPCs.

## Discussion

To mimic signals of the bone marrow ECM, three important features of the ECM have to be taken into consideration: (i) (bio)chemistry, (ii) mechanical properties and (iii) (nano)structure. In principle, all three properties can be controlled in the nanostructured hydrogel system we applied, making it highly suitable for the investigation of biomimetic cell-matrix interactions. In the current study we focused on ligand identity and nanostructure. We determined that nanostructured hydrogels biofunctionalized with different ECM-derived ligands, all of which are present in the bone marrow HSC niche, can influence the adhesive behavior of HSPCs and KG-1a cells depending on the ligand type and nanostructured presentation. Furthermore, THBS2 expression by HSPCs was regulated by the presence of a functional FN-derived ligand as well as the nanometer-scaled interligand distance. We identified THBS2 as an adhesive protein for human HSPCs.

To date the *in vivo* niche is the only environment known which allows HSC proliferation under full maintenance of their stem cell potential. Therefore, biomimetic approaches for culturing HSPCs are promising and are pursued with different approaches [53]. Besides investigating the impact of medium composition and ligand coatings on HSPCs, the influence of environmental parameters such as matrix stiffness [37], or scaffold microstructure on HSPC proliferation and differentiation have been investigated. For example, spatial restriction of HSPCs in ECM-coated microcavities (15 to 80 µm in diameter) has been shown to support the maintenance of immature HSPCs in quiescence [17]. With our study we addressed structural elements one scale length smaller – the nanometer scale. Our finding that HSPCs are sensitive to nanostructural surface features is in accordance with previous studies showing that the expansion and adhesion of HSPCs depend on the nanoscale topography of nanofiber substrates [42] and that adhesion and lipid raft clustering of HSPCs are influenced by the nanostructured presentation of cRGD-ligands on PEG-passivated glass surfaces [36].

We provide evidence that the nanometer-scaled lateral distance of several different ECM-derived ligands is an important determinant of HSPC adhesion. The critical maximum interligand distance at which HSPC adhesion was supported depended on the ligand type (cRGD and cLDV <45 nm, OPN ∼75 nm, FNRGD ∼110 nm). The observed differences in tolerated distances between the peptides and the protein domains might be due to ligand size or the presence of synergy sequences. The ligands (immobilized on the NPs) are flexible, rather than rigid, structures. The FNRGD domain, with an estimated length of 12–14 nm, might be able to “shorten” the distance between two NPs by bending towards each other, and thereby enabling integrins to cluster. In contrast, the peptide length of the cRGD peptide (estimated at ∼ 3.5 nm) limits their ability to converge and enable integrin binding. Additionally, the protein domains provide synergy sequences such as PHSRN that are able to enhance the adhesive capacity of the ligand [31].

The lateral cRGD distance sufficient for KG1a and HSPC adhesion was different to that found in previous studies using REF52-fibroblasts, 3T3-fibroblasts, MC3T3-osteoblasts and B16-melanocytes [50], [54], [55]. One possible explanation may be differences in the adhesive behavior of these cell types, related to whether they are anchorage-dependent (such as the cells used in former studies) or non-anchorage-dependent cells (such as the cells of the current study). Cell size, morphology and signaling vary greatly between the previously investigated anchorage-dependent and the non-anchorage-dependent hematopoietic cells and may contribute to differences in cell adhesion to nanopatterned surfaces. The molecular mechanisms underlying the cellular sensitivity to lateral ligand spacing at the nanometer scale (leading to differences in cell adhesion) are thought to depend on the composition and action of the protein network involved in the formation of the adhesion plaque and its interaction with the actin cytoskeleton [56]. While the integrin-mediated focal adhesion sites of anchorage-dependent cells such as fibroblasts and osteoblasts are well studied, relatively little is known about these adhesions in HSPCs. Nevertheless, it is clear that the integrin-linked multiprotein complexes of HSPCs and anchorage-dependent cells differ from each other, e.g., in the principal adhesion kinase, which is Pyk2 in HSPCs and FAK in anchorage-dependent cells [57], [58]. Furthermore, the actin cytoskeleton displays fundamental differences with its fibrous appearance in adherent and spreading anchorage-dependent cells and a ring-like structure in hematopoietic cells that keep more or less their round shape also upon adhesion to a solid support. Such differences in the composition of the integrin-linked multiprotein complex, the central signaling molecules such as kinases and the cytoskeleton, might cause the cell type dependence of the maximum tolerated ligand spacing. However, elucidating the mechanisms underlying the observed differences remains an ongoing challenge.

In our previous study using KG-1a cells and HSPCs, the maximum tolerated distance for adhesion to cRGD functionalized, nanostructured, PEG-passivated glass surfaces was determined at 32±6 nm [36]. In the present study, the maximum tolerated cRGD spacing for KG-1a cells on cRGD-functionalized PEG hydrogels was observed between 37±7 nm and 45±8 nm. Such variances may be a result of differences in the physical and chemical properties of the applied substrates, such as organization of the PEG. For the two different nanostructured surface types (passivated glass and hydrogel) passivation against unspecific protein adsorption and cell adhesion was achieved by using PEG as a background. However, different PEG molecules that vary for example in the chain length were used for the two surface types (M_r_ = 700 for hydrogels and M_r_ = 2000 for the PEG passivation layer on glass). These differences in chain length could lead to different molecular conformations of the PEG [59]. While the PEG molecules within the PEG monolayer on the glass are highly ordered, presenting a terminal methyl group at the surface [60], the PEG molecules in the hydrogels are randomly orientated, which means that on average all structural elements of the PEG molecules are exposed at the hydrogel surface to an equal extent. Such alterations in PEG molecules, orientation and cross-linking result in different chemical structures that are exposed to the cells. These chemical dissimilarities plus additional factors with the potential to influence cell adhesion such as the surface charge [61], hydrophobicity and surface roughness [62] might contribute to the slightly different HSPC adhesive behavior on nanostructured hydrogels and passivated glass surfaces. It was described previously that material stiffness influences cell-adhesion and stem cell proliferation, differentiation or self-renewal [37], [63], [64], [65]. We could recently show that HSPCs are mechanosensitive to hydrogels with Young’s moduli below 100 kPa [38]. This is in line with other reports on the biologically relevant stiffness range [63]. Since the Young’s moduli of the applied PEG hydrogels (6 MPa [46]) and of glass (64 GPa [66]) are much higher, and lie in a range in which cells are not mechanosensitive, we conclude that both substrate types appear very stiff to cells and that the mechanical properties do not contribute to the observed small differences. In summary, our adhesion studies demonstrate that the cellular sensitivity to nanometer scale lateral spacing depends on (i) the cell type, (ii) the targeted receptor and (iii) the provided ligand.

Hematopoietic cells were in contact with the nanostructured matrices via filopodia, but at the same time kept their round morphology. Because low conductivity and the swelling behavior of PEG hydrogels represent limitations for imaging cells on hydrogels by electron microscopy, higher resolution images were obtained on nanopatterned, PEG-passivated glass slides using scanning electron microscopy. The results show that cells were in contact with the immobilized cRGD ligands on the gold NP arrays, but did not bind to the PEG layer between the NPs. This is in line with previous findings for other cell types, e.g., fibroblasts [41].

HSPC adhesion to FN was mediated by a donor-dependent combination of α_4_β_1_ and α_5_β_1_ integrins. The integrin receptors α_4_β_1_ and α_5_β_1_ are important for mediating adhesion as well as for migration and homing of HSCs in the niche and for cell survival in co-culture with osteoblasts [67], [68]. It has been shown before that HSPC binding to FN is mediated through integrin α_4_β_1_ (which binds to the LDV motif) and through integrin α_5_β_1_ (which binds to the RGD motif) [69], [70]. In these studies integrins were additionally activated through antibodies or cytokines, which can both have an impact on cell adhesion. In the current study adhesion to FN was investigated only in the presence of ions that are necessary for integrin activation. However, our result differs from a previous study, in which HSPC adhesion to FN could be prevented by inhibiting only the integrin α5 chain in similar experiments with integrin blocking antibodies [15]. Possible explanations for this discrepancy might be differences in the study design including the immobilization of FN, the source and isolation of HSPCs (CD133^+^ cells from peripheral blood of G-CSF-treated healthy volunteers [15] and CD34^+^ cells from umbilical cord blood in the present study) and the individual variability from donor to donor.

Blocking the RGD recognition site by pre-incubation with RGD peptides was sufficient to completely block cell adhesion to FN. This indicates that the presence of the RGD molecule within FN is essential for HSPC adhesion. RGD also binds to α_4_β_1_ integrins, in addition to α_5_β_1_ integrins, at higher concentrations [71]. This may explain the complete loss of cell adhesion, including adhesion to the LDV motif.

The donor-dependent differences that we found when inhibiting cell adhesion to FN by blocking α_4_ or α_5_ integrin may be the result of different integrin expression levels in cells derived from different donors. In HSPCs isolated from human cord blood α_v_β_3_ integrin is not expressed or only expressed in very low amounts [38]. In accordance with this finding, we did not find evidence for α_v_β_3_ integrin involvement in HSPC adhesion to FN.

Immobilized FN-derived and OPN-derived ligands did not affect HSPC proliferation or differentiation. This is in contrast to previous publications that showed a negative influence of OPN on murine HSPC proliferation [33], [34]. One possible explanation for this discrepancy may be that the OPN constructs used in those studies were different to the thrombin-cleaved OPN fragment that we applied. Another possible explanation might be the intrinsic differences between the murine and the human system. Both positive and negative influences on HSPC proliferation have been described for FN [17], [25], [26], [27]. In these studies the full-length proteins were immobilized on substrates in random orientation, which is in contrast with our study where the FN domain type III 7–10 was offered in an oriented manner. Possible explanations for divergent findings concerning the influence of FN on cell proliferation are: (i) different surfaces (plastic, glass or gold) might lead to different conformations of FN, which can have an impact on cell proliferation and differentiation [28], (ii) differences in the orientation of the immobilized ligands and (iii) the availability of additional cell interaction sites on the full-length protein in comparison to the much shorter domain. Since, as we could show, α_5_β_1_ integrin mediates cell binding to the FN domain type III 7–10 and this domain does not affect HSPC differentiation, we conclude that in our setup α_5_β_1_ integrin had no impact on HSPC differentiation. This is in consensus with other publications showing that the loss of β_1_ integrin in HSCs does not influence differentiation into blood cells [22]. Yokota *et al.* did not find any influence of the FN domains type 8–10 on HSPC differentiation, which is also in good agreement with our findings [24].

Interestingly, we found that both THBS2 mRNA and protein are expressed by human HSPCs *in vitro*. We determined the presence of (or lack of) the RGD sequence within the FN domains type III 7–10 to influence THBS2 expression in adhering HSPCs, suggesting that THBS2 expression is regulated by α_5_β_1_ integrin-mediated signaling pathways. Integrin signaling induced by FN domain type III 7–10 led to a reduction in THBS2 expression. Furthermore, we could show that THBS2 expression depends on lateral ligand spacing and, in consequence, on lateral integrin clustering. THBS2 expression increased with increasing distance between FNRGD-functionalized NPs and the respective integrin receptors. Future investigations will reveal whether the defined lateral distances or a FN dose-dependent mechanism trigger THBS2 expression.

THBS2 is an ECM protein secreted by fibroblasts, osteoblasts and mesenchymal stem cells, and has, to our knowledge, not been described in HSCs as of yet [72], [73]. It acts as a matricellular protein that interacts with cell surface receptors such as integrins and with other extracellular components including matrix proteins, growth factors and proteases [74], [75]. THBS2 knockout mice showed connective tissue abnormalities, such as disordered collagen fibrillogenesis, abnormal bone formation with increased total density, cortical thickness of long bones, and a reduced marrow cavity [76]. THBS2 has been shown to inhibit marrow stromal cell proliferation, which indicates a function in the bone marrow microenvironment [72].

In the present study, we show that HSPCs can express THBS2 and are able to adhere to it. This indicates that THBS2 is involved in HSPC regulation and that HSPCs can influence their ECM composition. Furthermore, HSPC adhesion to THBS2 suggests that, in addition to having paracrine effects on the surrounding cells and matrix, THBS2 also has autocrine effects on HSPCs themselves. One possible explanation for the FN-dependent THBS2 expression pattern might be that HSPCs produce the matrix protein in the absence of or at low FN concentrations (such as on a non-adhesive PEG-background), in order to generate a surrounding matrix. Since THBS2 can interact with other ECM proteins to organize the ECM and can also influence other niche cells such as mesenchymal stem cells [72] and osteoblasts [77], it is likely that HSPCs can actively modulate their environment. This hypothesis is supported by previous findings, which describe a regulation of the niche by HSPCs [2], [3]. Here we report that HSPCs can directly influence the composition and organization of the ECM, and thereby have the ability to modulate the niche by a mechanism that functions independently of cell-cell contacts.

In conclusion, we show that HSPCs are sensitive to the nanostructured presentation of several ECM derived ligands. Although no influence of FN-derived or OPN-derived ligands on HSPC proliferation and differentiation was observed, THBS2 was expressed by HSPCs in an integrin-mediated, ligand-dependent and nanostructure-dependent manner. In addition, THBS2 mediates HSPC adhesion. Thus, we conclude that under unfavorable conditions, where ligands needed for adhesion are absent or spread too far apart from each other, HSPCs are able to modulate their environment and actively participate in the formation and regulation of the HSC niche.

## Materials and Methods

### Nanopatterning

Glass slide nanopatterning by block-copolymer micelle nanolithography (BCML) was performed as previously described [45], [78]. With this technique quasi-hexagonally ordered nanoparticle arrays with lateral distances between gold NPs ranging from 20±6 to 110±18 nm were produced. The parameters of the nanopatterning processes for the different surfaces are given in Table 1. The gold nanopatterns were transferred to PEG diacrylate (M_r_ 700; PEG 700 DA) hydrogels according to a protocol published previously [46], [79]. The applied PEG 700 DA hydrogels exhibit a Young’s modulus of 6 MPa [46]. For high resolution microscopy, glass substrates were passivated with a protein repellent layer of PEG triethoxysilane that prevents unspecific protein adsorption to the glass [60], as described recently [36].

**Table 1:** Parameters for producing nanostructured surfaces by BCML.

PS(x)-b-P2VP(y)	C *[mg/ml]*	V *[mm/min]*	d *[nm]*
PS(154)-*b*-P2VP(33)	5	24	20±6
PS(240)-*b*-P2VP(143)	5	24	30±6
	5	18	35±7, 36±7 or 37±7
	4	10	45±8
PS(1056)-*b*-P2VP(495)	5	30	60±11
	5	24	65±11
	4	24	75±13
	4	12	85±14
	3	16	110±18

PS: polystyrene units, P2VP: poly(2-vinylpyridine) units, C: concentration of the polymer, V: substrate retraction velocity, d: distance between gold NPs ± standard deviation.

### Protein Expression and Purification of Recombinant Proteins

The pET15b plasmids encoding for the fibronectin domain type III 7–10 with the RGD sequence (FNRGD) and the deletion mutant (FNΔRGD) were kindly provided by Prof. Dr. R. Fässler (Max Planck Institute for Biochemistry, Martinsried, Germany). A pET15b plasmid encoding amino acids 17–168 of osteopontin (the N-terminal thrombin fragment, hitherto referred to as shortened OPN or OPNs) was obtained from GenScript (Piscataway, NJ, USA). The *E.coli* strain BL21 (Invitrogen) was used for protein expression, and expression was induced according to a protocol described by Studier [80]. Bacterial cell pellets were resuspended in PBS and lysed by repeated freezing and thawing cycles and ultrasonic sound treatment. The N-terminal His-tagged fibronectin type III 7–10 (containing the RGD sequence) and the deletion mutant FNΔRGD as well as OPNs were isolated and purified using a HisTrap™FF chromatography column containing precharged Ni-sepharose (GE Healthcare, Uppsala, Sweden) according to the instructor’s manual. Eluates were desalted using PD-10 desalting columns (GE Healthcare) to remove all imidazole compounds and finally eluted with PBS. The purity of all recombinant proteins was controlled by silver stain analysis.

### Biofunctionalization

Nanostructurally predefined hydrogels were functionalized with short peptide motifs (cLDV, cRGD or cRGE – a non-adhesive control peptide), larger protein domains (the N-terminal thrombin cleaved fragment of osteopontin or a cell-binding fibronectin domain containing the type III modules 7–10– the latter domain with or without the crucial RGD sequence) and full-length fibronectin. Details are given in Table 2. Prior to functionalization, nanostructured hydrogels were sterilized in 70% ethanol and washed. Biofunctionalization with FN-derived short peptide motifs (25 µM in aqueous solution) was achieved by incubating at room temperature for 2 h. Functionalization with His-tagged recombinant protein domains (12.5 µM solution in PBS) was accomplished using an NTA-thiol linker system, as described elsewhere [51], [81]. Hydrogels were washed rigorously with PBS (3 times for 20 min) to remove unbound ligands. Before cell experiments were performed, biofunctionalized hydrogels were equilibrated for 30 min in cell-specific media under standard cell culture conditions.

**Table 2:** Short peptide binding motifs and protein domains used for biofunctionalization of gold NP arrays.

Name	Sequence/Description	Molecular weight
cRGD	Cys-PEG_6_-Nε(Lys-Arg-Gly-Asp-D-Phe)_cyclo_	1042 Da
cRGE	Cys-PEG_6_-Nε(Lys-Arg-Gly-Glu-D-Phe)_cyclo_	1056 Da
cLDV	Cys-PEG_6_-Nε(Lys-Leu-Asp-Val-D-Phe)_cyclo_	1046 Da
FNRGD	FN domain type III modules 7–10 N-terminal His-tag	40077 Da
FNΔRGD	FN domain type III modules 7–10 ΔRGD N-terminal His-tag	39731 Da
OPNs	OPN domain (amino acid 17–168) N-terminal His-tag	19426 Da

Peptides were purchased from Biosyntan, Berlin, Germany or synthesized by Dr. Hubert Kalbacher (University of Tübingen, Germany).

Full-length FN protein was isolated from human plasma as described elsewhere [82] and adsorbed to nanostructured hydrogels for relative comparison purposes.

### Immunofluorescence Staining of Hydrogels

Hydrogels functionalized with FN domains (FNRGD or FNΔRGD) were incubated with 10 µg/ml mouse-anti-human FN (Clone FN12-8, QED Bioscience Inc., San Diego, USA) overnight at 4°C. After washing with PBS the hydrogels were incubated with a secondary goat-anti-mouse IgG1 Alexa Flour 488 antibody (Invitrogen, Darmstadt, Germany) for 45 min. Because EDTA forms a complex with Ni^2+^ and other cations necessary for successful formation of NTA-His-tag complexes, one control was incubated with 25 µM EDTA instead of nickel chloride, hindering protein coupling via the His-tag. A second control was incubated without the primary antibody to test for unspecific antibody binding. The hydrogels were imaged using an Axiovert 200 M microscope (Zeiss, Jena, Germany).

### Cell Culture

The human acute myeloid leukemia cell line KG-1a (DSMZ, Braunschweig, Germany), cultured in RPMI, 20% FBS (Invitrogen or Sigma, Taufkrichen, Germany) and 1% (v/v) penicillin/streptomycin (P/S, Gibco, Darmstadt, Germany) under standard cell culture conditions, was used as a model cell line for immature hematopoietic cells. Primary human CD34^+^ HSPCs were isolated from umbilical cord blood with a CD34 magnetic bead system (Miltenyi Biotec, Bergisch-Gladbach, Germany) according to the manufacturer’s instructions and maintained in SFEM Media supplemented with 1% (v/v) cytokine mix cc100 (both Stemcell Technologies, Grenoble, France) and 1% (v/v) P/S at 37°C and 5% CO_2_. Purity of the isolated cells was controlled by flow cytometry using CD34-PC5 or PC7 antibodies (Clone 581, Beckman Coulter, Krefeld, Germany) and the respective isotype controls (Beckman Coulter) on a Cytomics FC500 flow cytometer (Beckman Coulter). They were only used if more than 95% of the cells were CD34 positive.

### Ethics Statement

Umbilical cord blood was obtained from the DKMS umbilical cord blood bank in Dresden and the University Hospital of Tübingen after written and informed consent of the parents and approval by the local ethics committee (Ethik-Kommission der Medizinischen Fakultät und am Universitätsklinikum Tübingen, project numbers 120/2012BO2 and 005/2012B02).

### Scanning Electron Microscopy (SEM) of Cells on Nanostructured Surfaces

Adherent cells on PEG hydrogels or PEG-passivated glass slides were fixed with 4% paraformaldehyde (PFA; Alfa Aesar, Karlsruhe, Germany) in PBS for 20 min at room temperature. The aqueous liquid was exchanged with ethanol in a series of washing steps with increasing ethanol concentrations. The samples were critical point dried (Critical Point Drying Device Leica EM CPD030 Leica Microsystems, Wetzlar, Germany), coated with carbon (Modular High Vacuum Coating System MED 020, Leica Microsystems) and sample images were taken with an Ultra 55 field emission electron microscope (Zeiss). Hydrogel cryo SEM images were obtained under low temperature conditions (T ≈ −130°C). To cool the samples in liquid nitrogen and transfer them into the SEM chamber a BAL-TEC VLC 100 (BAL-TEC AG, Balzers, Lichtenstein) shuttle and a BAL-TEC MED 020 (BAL-TEC AG) loading device were used.

### Cell Adhesion to Nanostructured Surfaces

2×10^6^ KG1-a cells were incubated on biofunctionalized hydrogels in adhesion medium [RPMI supplemented with an ion mix (final concentration 1 mM CaCl_2_, 1 mM MgCl_2_, 25 µM MnCl_2_)] at 37°C and 5% CO_2_ for 1 h. The ions are essential for integrin activation [83]. After washing the hydrogels twice with PBS, surface images were taken at the interface between the nanostructured and the unstructured area using an Axiovert 40 CFL microscope (Zeiss).

Quantification of cell adhesion to different ligands on differently nanostructured hydrogels required measurements in a multiwell format. For this purpose, the nanostructured hydrogels were produced identical in size to a standard 24 mm x 60 mm microscope slide. The surface of these gels was divided into different growth areas (each one identical in size to a well of a 96-well plate) by using the flexiPERM system (Greiner Bio-One, Frickenhausen, Germany). After biofunctionalization and a washing step, 2×10^4^ KG-1a cells or 1×10^4^ freshly isolated HSPCs suspended in adhesion medium were added per well and incubated for 1 h at 37°C and 5% CO_2_. Nonadherent cells were carefully removed with the medium and the remaining adherent cells were stained using the Cy Quant NF Cell Proliferation Assay Kit (Invitrogen). Fluorescence intensity, which is proportional to the number of stained cells, was detected at 480 nm. The relative fluorescent intensity of each sample was normalized to the value obtained for cell adhesion to the adsorbed full-length FN protein for comparison. The fluorescence intensity of wells with cells on unstructured, not functionalized hydrogels was set as background fluorescence and subtracted from all other values.

### Integrin Inhibition Adhesion Assay

One drop (1 µl) of 1 mg/ml FN was allowed to air dry on a tissue culture plastic dish. Unspecific cell binding to the dish was prevented by blocking with 1% BSA (albumin bovine fraction V, Serva Electrophoresis, Heidelberg, Germany) in PBS for 1 h and washing with PBS. For integrin inhibition assays, HSPCs were pre-cultured for 20 h, washed with PBS and incubated with the respective antibodies for 1 h at 37°C (details on the applied antibodies can be found in Table 3). Thereafter, the cells were distributed on to the prepared surfaces and incubated in adhesion medium for 1 h at 37°C and 5% CO_2_. Finally, the dishes were carefully rinsed 3 times with PBS to remove unbound cells. The remaining adherent cells were imaged (Axiovert 40 CFL microscope). The cell number on each FN spot was determined by manual counting.

**Table 3:** Antibodies for integrin inhibition assays.

Antibody	Clone	Concentration	Supplier
Mouse-anti-human CD29-RD1 (β_1_ integrin)	4B4LDC9LDH8	1∶20	Beckman Coulter
Mouse-anti-human CD49d (α_4_ integrin)	2B4	5 µg/ml	R&D Systems
Goat-anti-human CD49e (α_5_ integrin)	polyclonal	20 µg/ml	R&D Systems
IgG1-PE (isotype control)	679.1Mc7	1∶5	Beckman Coulter

### Thrombospondin Adhesion Assay

To quantify cell adhesion to THBS2 a 96-well plate was coated with 10 µg/ml THBS2 (recombinant human THBS2, R&D Systems, Wiesbaden, Germany), 10 µg/ml FN or BSA and incubated for 12 h at 4°C. To enhance its adhesive activity, THBS2 was reduced with 20 mM dithiothreitol [84] in PBS for 20 min, followed by 3 washing steps. 2.5×10^4^ HSPCs (pre-cultured for 20 h) were applied per well and incubated for 1 h at 37°C and 5% CO_2_ in adhesion media. The wells were carefully washed with PBS and the remaining adherent cells were stained using the Cy Quant NF Cell Proliferation Assay Kit, as described above. The reference value (100% value) was determined by staining 2.5×10^4^ HSPCs.

### Proliferation

A 5 nm adhesive titanium layer was sputtered on a glass coverslip using the Modular High Vacuum Coating System MED 020 (Leica Microsystems). An additional 50 nm gold layer was sputtered on top. Biofunctionalization of these continuous gold surfaces (same procedure as described for nanostructured substrates) resulted in the densest achievable packing of ligands. Isolated HSPCs were stained with CFSE (3.5 µM) for 10 min at 37°C in PBS with 0.1% FBS. Staining was stopped by incubating in PBS with 10% FBS for 5 min on ice. After washing with PBS, cells were cultured on the prepared sterilized surfaces in SFEM medium with 1% (v/v) cytokine mix cc100 and 1% (v/v) P/S. After 4 and 7 days cells were counted and stained with CD34-PC7 or IgG1-PC7 antibodies (Beckman Coulter, Krefeld, Germany) for 30 min on ice and analyzed with a FC 500 flow cytometer (Beckman Coulter).

### Determination of HSPC Differentiation Using the Colony-forming Cell Assay

After the proliferation assay, an aliquot of the *in vitro* proliferated HSPC was subjected to differentiation analysis by colony-forming cell assay. This assay allows the enumeration and classification of formed colonies according to their morphology. 1500 cells were washed, resuspended in 300 µl IMDM media and vortexed with 3 ml MethoCult H4434 Classic methylcellulose-based Media (both Stemcell Technologies) supplemented with 1% (v/v) P/S. 1.1 ml of this mixture were plated in triplicates in 35 mm petri dishes and incubated for 13 days at 37°C and 5% CO_2_. The different types of colonies were determined according to the “Atlas of human hematopoietic colonies from cord blood” (Stemcell Technologies) using an Axiovert 40 CFL microscope.

In order to study the influence of nanostructurally presented ligands on HSPC differentiation 500 freshly isolated HSPCs in 100 µl IMDM media were applied onto nanostructured, functionalized PEG hydrogels in 35 mm petri dishes and incubated for 1 h at 37°C and 5% CO_2_ in a wet-chamber. Then 1 ml MethoCult H4434 classic methylcellulose-based media supplemented with 1% (v/v) P/S was added and the plates were incubated for 13 days at 37°C and 5% CO_2_.

### Real-time Reverse Transcription Polymerase Chain Reaction (qRT-PCR)

2×10^5^ freshly isolated HSPCs were incubated on nanostructured, biofunctionalized hydrogels for 140 min in SFEM supplemented with 1% (v/v) P/S and the ion mix (1 mM CaCl_2_, 1 mM MgCl_2_, 25 µM MnCl_2_) at 37°C and 5% CO_2_. After removing the cells from the hydrogels, mRNA was isolated using the RNeasy MicroKit (Qiagen, Hilden, Germany) according to the manufacturer’s protocol. 40 ng mRNA were reverse transcribed into cDNA applying the High Capacity cDNA Synthese Kit (Applied Biosystems, Darmstadt, Germany). After 10 cycles of cDNA pre-amplification using the TaqMan PreAmp Master Mix Kit (Applied Biosystems), quantitative real time PCR was performed with the TaqMan system and comparative ΔΔCT method (Applied Biosystems 7500 System). The relative quantifiation (RQ) values were calculated using the 7500 v2.0.1 software (Applied Biosystems; RQ = 2^−ΔΔCt^) and give the change in expression of the test sample relative to the calibrator sample (fold change). Primers and probes for human THBS2 (Hs011568063_m1) and the endogenous control human beta-2-microglobulin (B2M) were purchased from Applied Biosystems. The relative THBS2 gene expression was normalized to the value obtained for cells incubated on unstructured, unfunctionalized hydrogels. Undetectable gene expression was set to 0. To exclude unspecific gene amplifications, the size of the PCR product was controlled by agarose (Biozym, Hessisch Oldendorf, Germany) gel electrophoresis.

### Immunofluorescence Staining of HSPCs

After incubation of HSPCs on nanostructured, biofunctionalized hydrogels for 13 h in SFEM supplemented with 1% (v/v) P/S and the ion mix at 37°C and 5% CO_2_, the cells were removed from the hydrogels, centrifuged and resuspended in PBS. The cells were spinned onto a microscope slide using a cytospin (Cellspin II, Thermac, Waldsolms, Germany) and fixed with 4% PFA. After washing with PBS and permeabilisation with 0.1% Triton X100 (Carl Roth, Karlsruhe, Germany), the cells were stained with 2.5 µg/ml mouse-anti-human THBS2 antibody (clone 230927, R&D Systems) for 2 h, washed with PBS and incubated with 2.5 µg/ml secondary antibody goat-anti-mouse IgG1 Alexa Fluor 488 for 1 h. After washing with PBS, slides were sealed with mounting media (Prolong Gold antifade reagent with DAPI, Life Technologies, Darmstadt, Germany) and imaged with the Axiovert 200 M microscope. The fluorescence intensity of THBS2 (Alexa Flour 488) was analyzed with Image J Software (http://rsb.info.nih.gov/ij/ National Institutes of Health, USA). The fluorescence of the negative controls (stained with the secondary antibody only) was set as background fluorescence and subtracted from the other values.

### Statistical Analyses

Each experiment was independently repeated 3 to 5 times. For statistical analyses the Wilcoxon rank sum test or the two-tailed unpaired Student’s t-test were performed using the MATLAB (The MathWorks, Inc, Natick, MA, USA) software and Microsoft Office Excel (Redmond, WA, USA), respectively. P values were regarded as statistically significant when smaller than 0.05. The type of test, exact numbers of independent experiments (N) and technical replicates in each experiment are given in the respective figure legends.

## Supporting Information

Figure S1 **Schematic representation of FN and FN-derived ligands applied in the present study.** (A) Modular organization of the FN monomer: FN contains type I (green), type II (red) and type III (magenta) modules. The variable region containing the LDV sequence is shown in blue. The FN type III domains 7–10 containing the RGD and the PHSRN (a cell-binding domain that activates integrins) sequences are enlarged. The FN type III 7–10 domain carries an N-terminal His-tag for biofunctionalization purposes. (B) The cRGD peptide with a PEG linker and a terminal cysteine. The thiol group of the cysteine side chain binds to a gold NP during biofunctionalization.
(TIF)

Figure S2 **Cell morphology on nanostructured PEG hydrogels.** SEM images of critical point dried KG-1a cells on nanostructured, cRGD-functionalized hydrogels with interparticle distances of 30±6 nm. Magnification increases from A to C.
(TIF)

Figure S3 **KG-1a cell adhesion to cRGD functionalized hydrogels with different nanoparticle distances.** Microscopic images of the border between the structured (bottom) and the unstructured (top) part of the nanostructured, cRGD functionalized hydrogels are shown. The distances between the gold NP on the different substrates are depicted above the pictures. Cells can be observed as bright spots on a grey background. Scale bar = 200 µm.
(TIF)

Figure S4 **Microscopic images of KG-1a cell adhesion to nanostructured hydrogels**. The hydrogels were biofunctionalized with (A) FNRGD and (B) OPNs protein domains. NP distances are indicated above the panels. The images were taken at the border between the structured and the unstructured part of the substrates. One of 5 (A) or 3 (B) representative experiments is shown. Scale bar = 200 µm.
(TIF)

Figure S5 **Microscopic images of HSPC adhesion to FNRGD spots.** Adhesion to the FNRGD domain (left) was inhibited by addition of a function-blocking β1 integrin antibody (right). Cells appear as bright spots on a dark background.
(TIF)

Figure S6 **HSPC differentiation on nanostructured hydrogels.** Differentiation of HSPCs on nanostructured hydrogels (37 nm) functionalized with two different peptide ligands. N_independent experiments_ = 3, error bars = standard deviation of the mean.
(TIF)

Figure S7 **HSPC proliferation assays.** (A) Cell proliferation was measured on day 4 and day 7 using a CFSE assay and is expressed as percentage in relation to the proliferation on unfunctionalized gold control surfaces. (B) The percentage of CD34 positive cells was determined after HSPC incubation for 4 or 7 days on glass slides biofunctionalized with different ligands. (C) Representative histograms of flow cytometry analyses of CFSE labeled cells after 4 days incubation on biofunctionalized glass surfaces. The respective ligands are named in the top left corner of each histogram and the number of cell divisions is indicated by vertical, dashed lines. (D) CD34 expression of HSPCs after 4 (red curve) and 7 (blue curve) days of incubation on biofunctionalized glass surfaces; The CD34 isotype control is shown in gray. N_independent experiments_ = 4; error bars = standard deviation of the mean; gold = homogeneous gold film on glass; FNΔRGD is abbreviated with “ΔRGD”.
(TIF)

Figure S8 **Immunofluorescence THBS2 staining of HSPCs.** Representative microscopic images of HSPCs incubated for 13 h on nanostructured, biofunctionalized hydrogels. The top row of images shows bright field images, in the middle row THBS2 is made visible by Alexa Fluor 488 fluorescence staining (green), and in the bottom row cell nuclei are made visible by Dapi staining (blue). The negative control was incubated without the primary antibody. One representative experiment (based on one donor) of 3 is shown. 20 cells per donor were analyzed on each substrate and one cell per substrate is shown. Scale bar = 10 µm.
(TIF)
