# Supplementary figures and images for: Regulation of Hematopoietic Stem Cell Behavior by the Nanostructured Presentation of Extracellular Matrix Components
Source: PLoS One. 2013 Feb 6;8(2):e54778. doi: 10.1371/journal.pone.0054778 (PMC3566109; doi:10.1371/journal.pone.0054778)

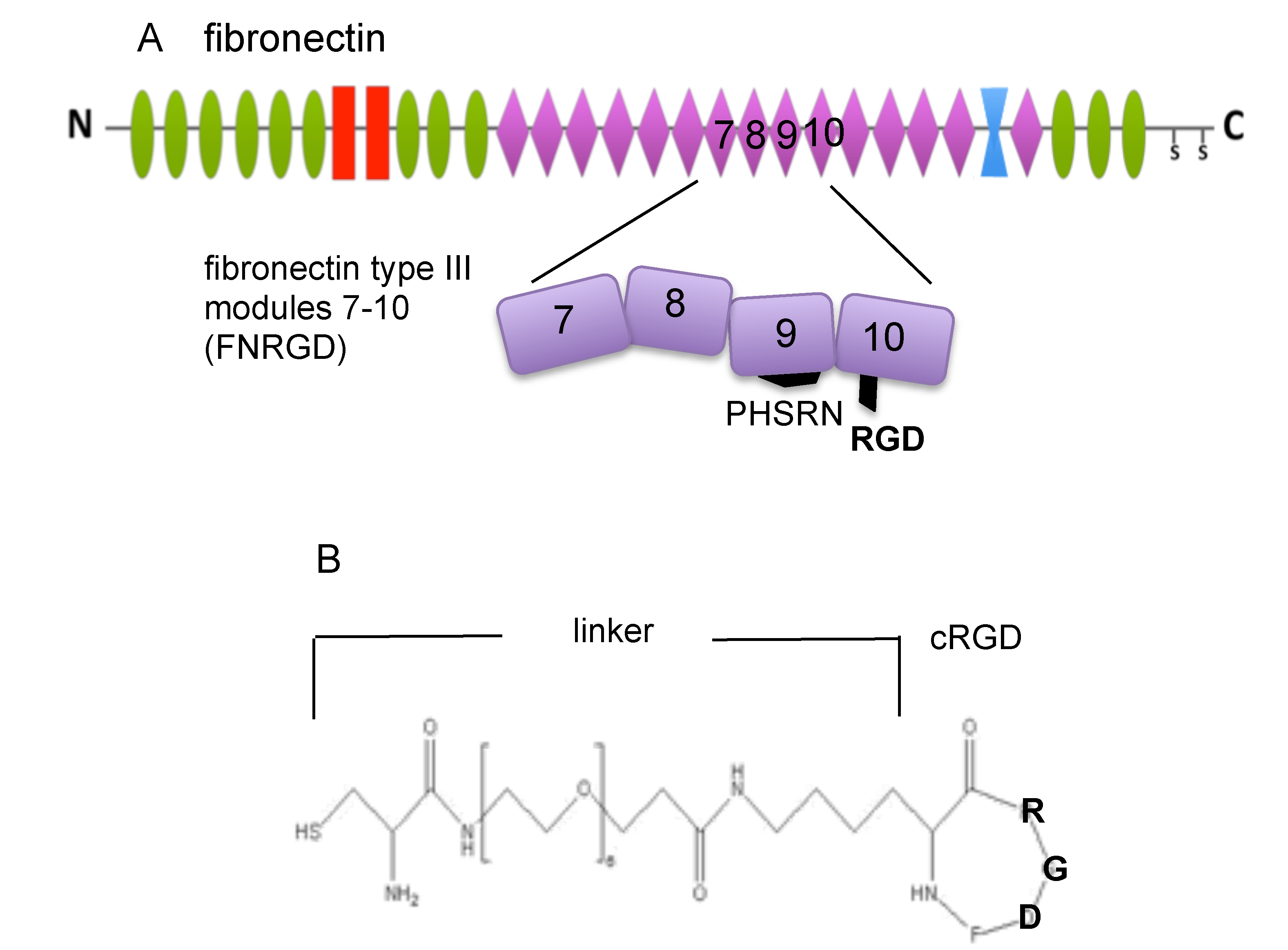

Supplement: Figure S1 — Schematic representation of FN and FN-derived ligands applied in the present study. (A) Modular organization of the FN monomer: FN contains type I (green), type II (red) and type III (magenta) modules. The variable region containing the LDV sequence is shown in blue. The FN type III domains 7–10 containing the RGD and the PHSRN (a cell-binding domain that activates integrins) sequences are enlarged. The FN type III 7–10 domain carries an N-terminal His-tag for biofunctionalization purposes. (B) The cRGD peptide with a PEG linker and a terminal cysteine. The thiol group of the cysteine side chain binds to a gold NP during biofunctionalization. (TIF) [file pone.0054778.s001.tif]

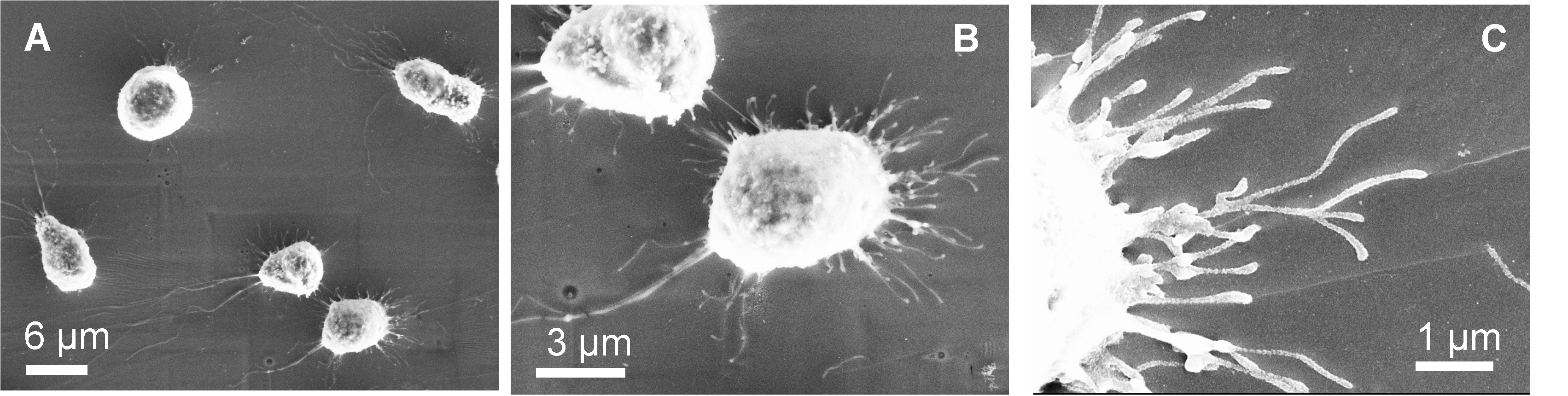

Supplement: Figure S2 — Cell morphology on nanostructured PEG hydrogels. SEM images of critical point dried KG-1a cells on nanostructured, cRGD-functionalized hydrogels with interparticle distances of 30±6 nm. Magnification increases from A to C. (TIF) [file pone.0054778.s002.tif]

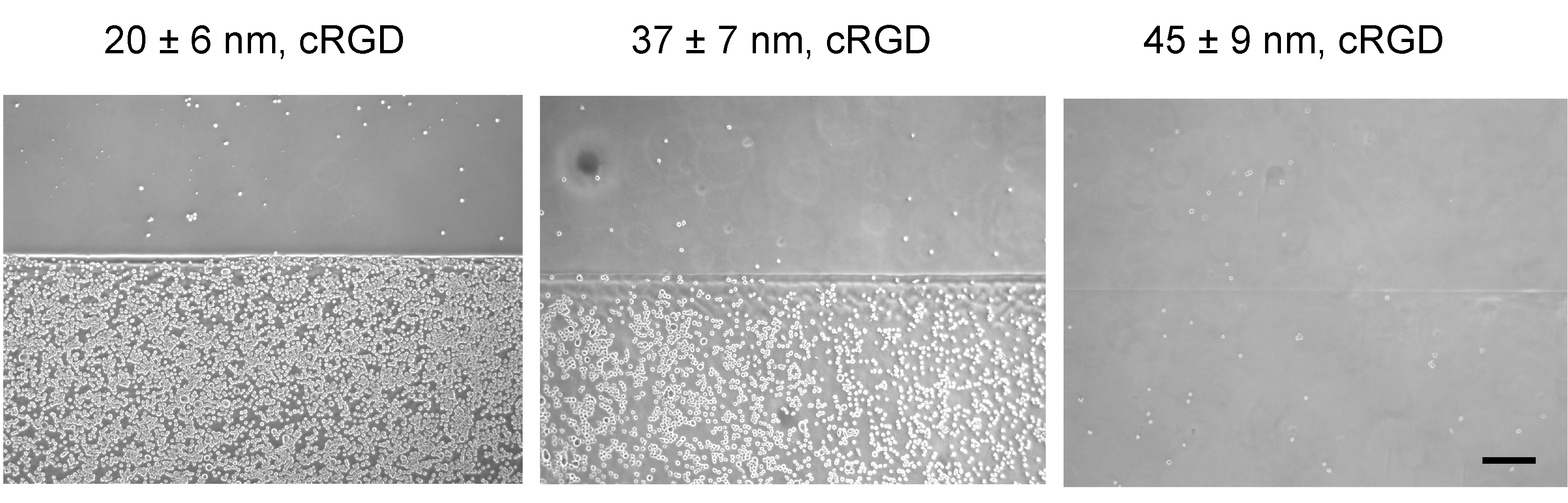

Supplement: Figure S3 — KG-1a cell adhesion to cRGD functionalized hydrogels with different nanoparticle distances. Microscopic images of the border between the structured (bottom) and the unstructured (top) part of the nanostructured, cRGD functionalized hydrogels are shown. The distances between the gold NP on the different substrates are depicted above the pictures. Cells can be observed as bright spots on a grey background. Scale bar = 200 µm. (TIF) [file pone.0054778.s003.tif]

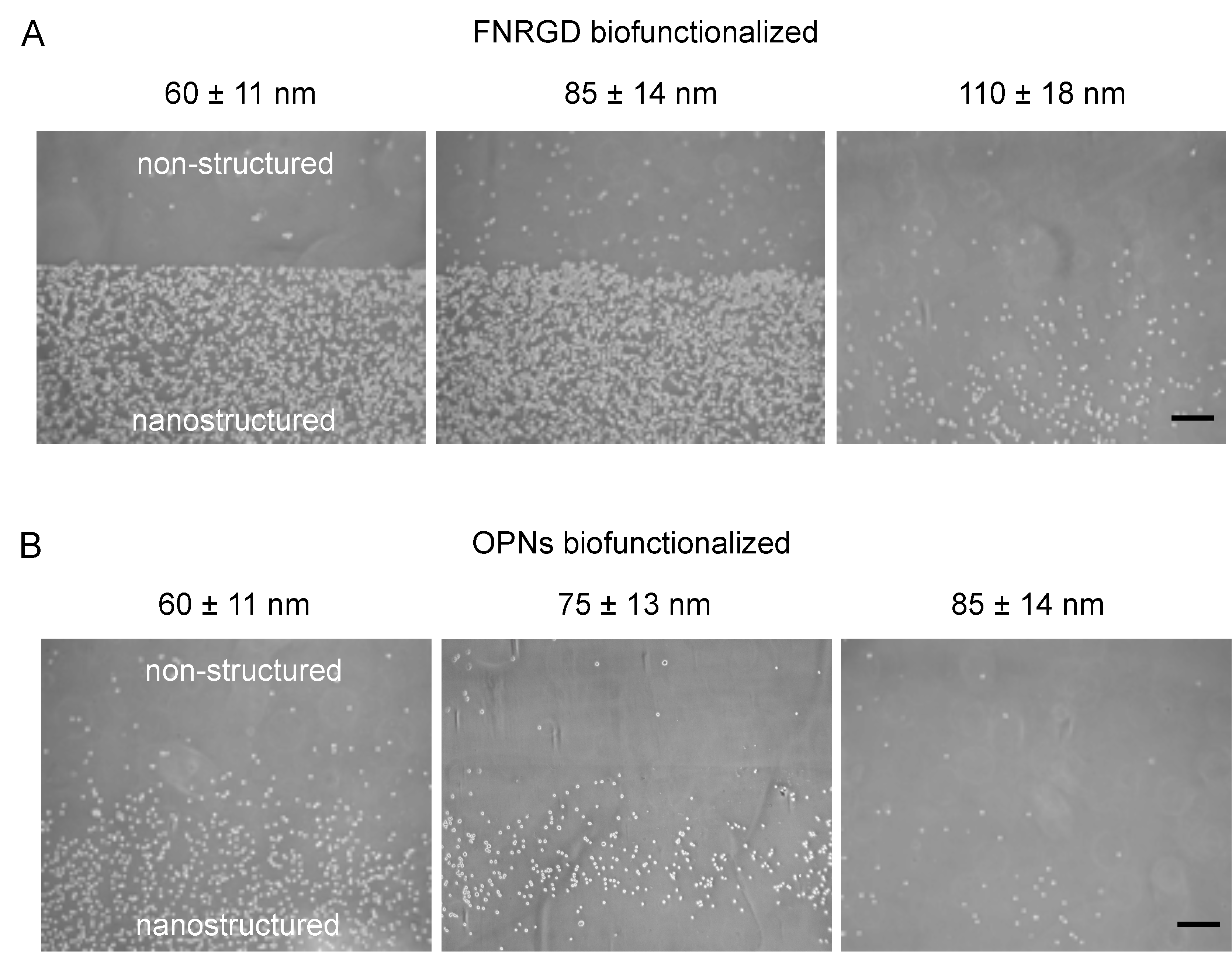

Supplement: Figure S4 — Microscopic images of KG-1a cell adhesion to nanostructured hydrogels. The hydrogels were biofunctionalized with (A) FNRGD and (B) OPNs protein domains. NP distances are indicated above the panels. The images were taken at the border between the structured and the unstructured part of the substrates. One of 5 (A) or 3 (B) representative experiments is shown. Scale bar = 200 µm. (TIF) [file pone.0054778.s004.tif]

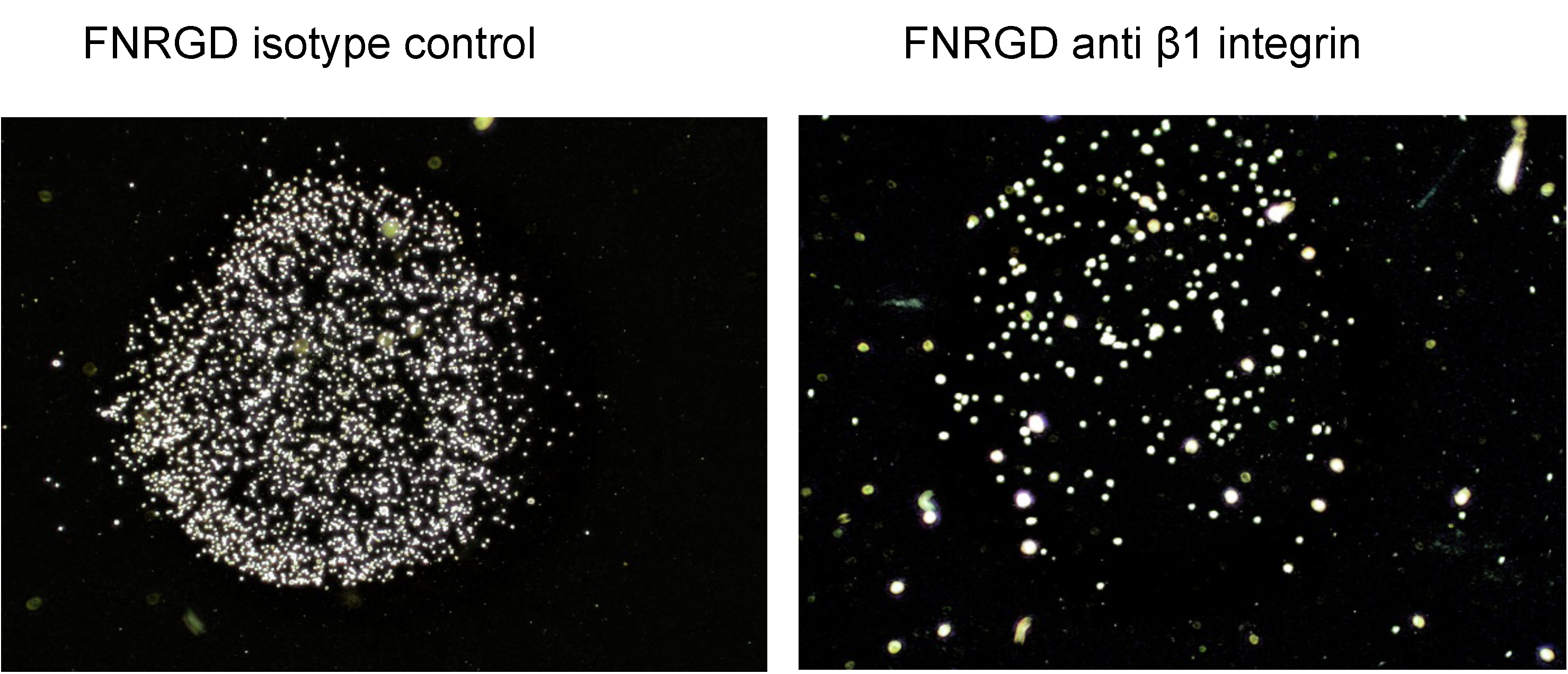

Supplement: Figure S5 — Microscopic images of HSPC adhesion to FNRGD spots. Adhesion to the FNRGD domain (left) was inhibited by addition of a function-blocking β1 integrin antibody (right). Cells appear as bright spots on a dark background. (TIF) [file pone.0054778.s005.tif]

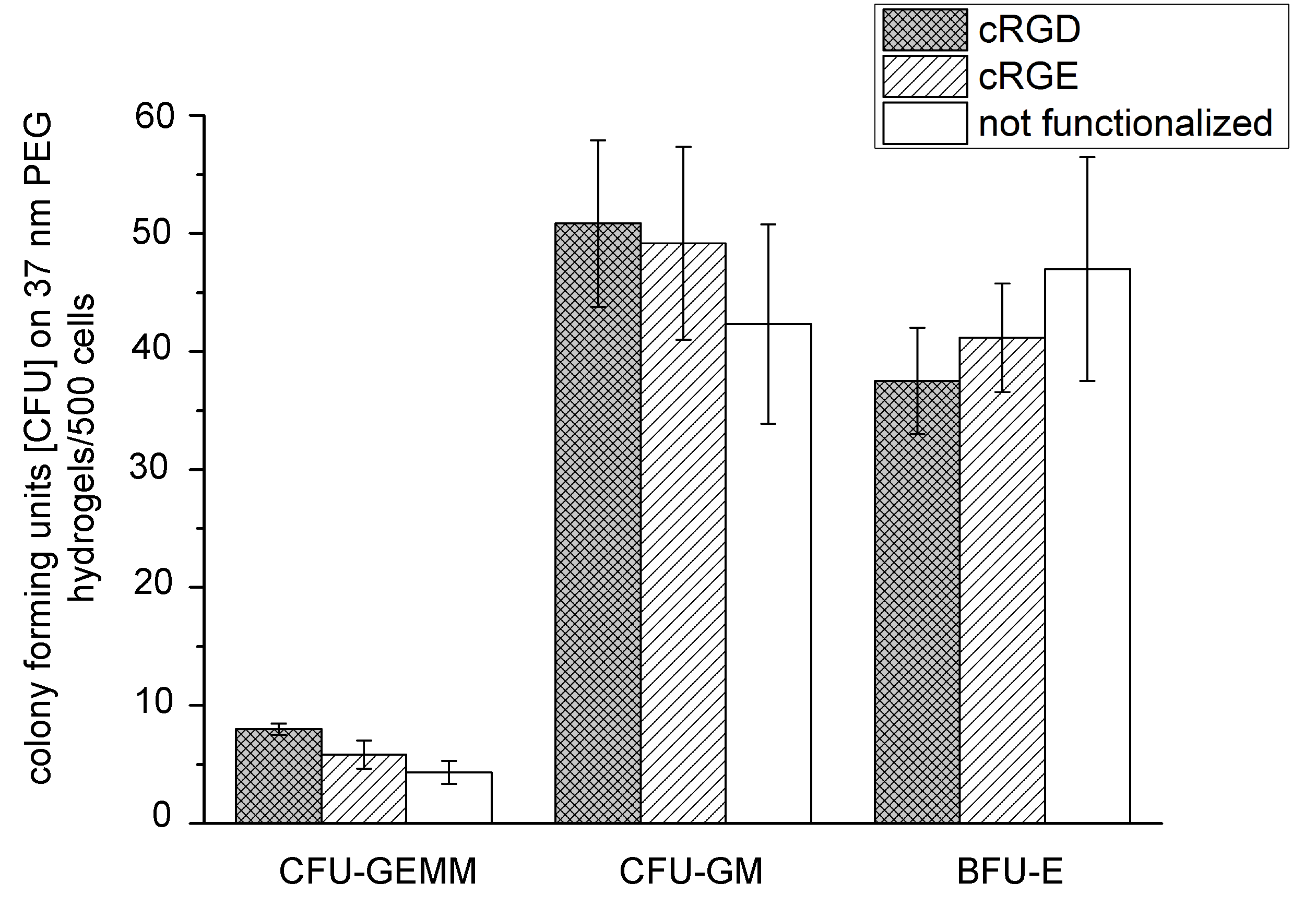

Supplement: Figure S6 — HSPC differentiation on nanostructured hydrogels. Differentiation of HSPCs on nanostructured hydrogels (37 nm) functionalized with two different peptide ligands. Nindependent experiments = 3, error bars = standard deviation of the mean. (TIF) [file pone.0054778.s006.tif]

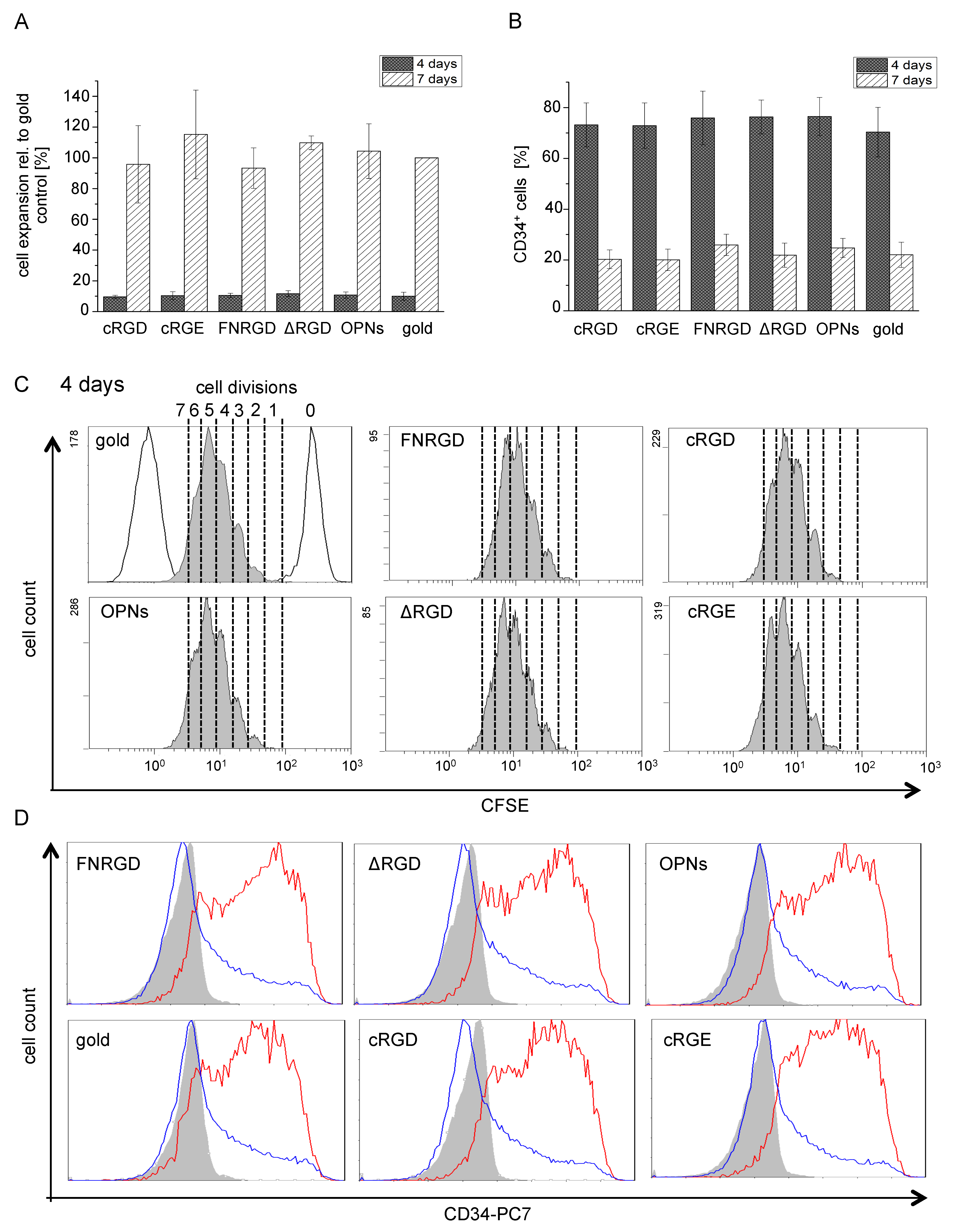

Supplement: Figure S7 — HSPC proliferation assays. (A) Cell proliferation was measured on day 4 and day 7 using a CFSE assay and is expressed as percentage in relation to the proliferation on unfunctionalized gold control surfaces. (B) The percentage of CD34 positive cells was determined after HSPC incubation for 4 or 7 days on glass slides biofunctionalized with different ligands. (C) Representative histograms of flow cytometry analyses of CFSE labeled cells after 4 days incubation on biofunctionalized glass surfaces. The respective ligands are named in the top left corner of each histogram and the number of cell divisions is indicated by vertical, dashed lines. (D) CD34 expression of HSPCs after 4 (red curve) and 7 (blue curve) days of incubation on biofunctionalized glass surfaces; The CD34 isotype control is shown in gray. Nindependent experiments = 4; error bars = standard deviation of the mean; gold = homogeneous gold film on glass; FNΔRGD is abbreviated with “ΔRGD”. (TIF) [file pone.0054778.s007.tif]

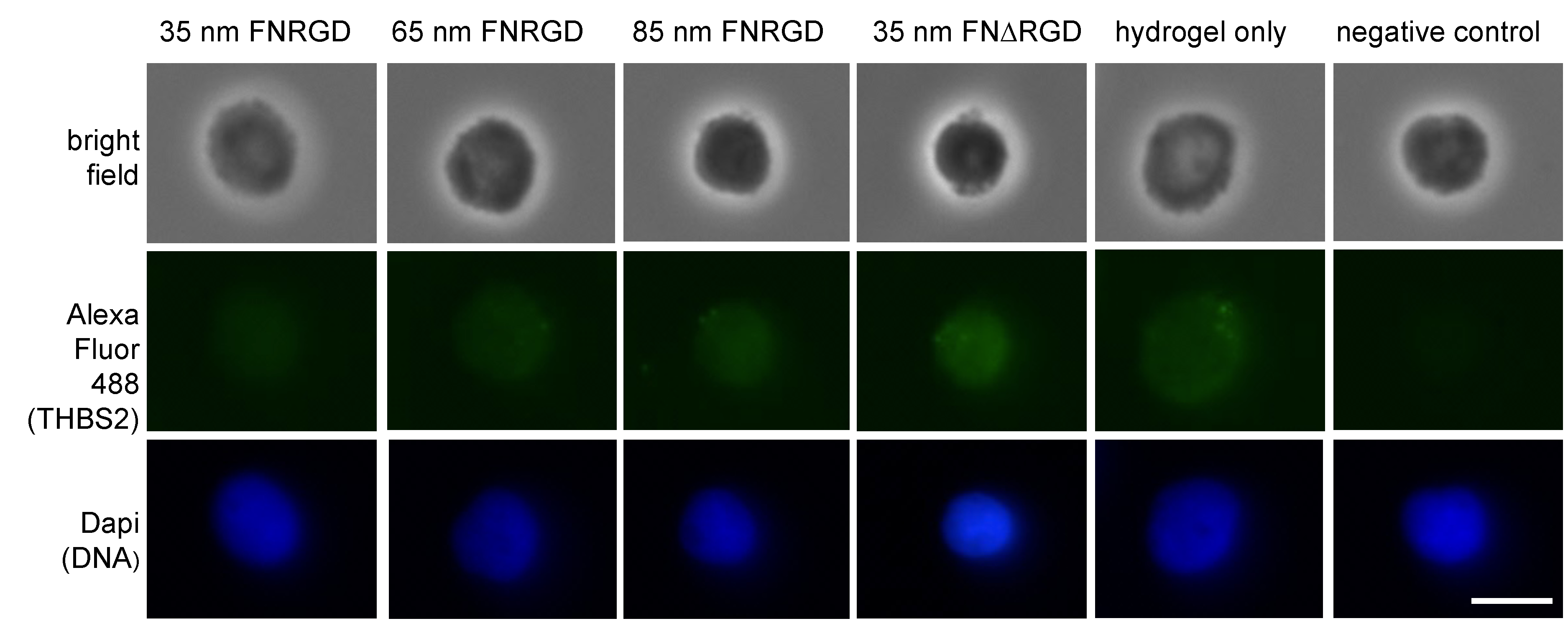

Supplement: Figure S8 — Immunofluorescence THBS2 staining of HSPCs. Representative microscopic images of HSPCs incubated for 13 h on nanostructured, biofunctionalized hydrogels. The top row of images shows bright field images, in the middle row THBS2 is made visible by Alexa Fluor 488 fluorescence staining (green), and in the bottom row cell nuclei are made visible by Dapi staining (blue). The negative control was incubated without the primary antibody. One representative experiment (based on one donor) of 3 is shown. 20 cells per donor were analyzed on each substrate and one cell per substrate is shown. Scale bar = 10 µm. (TIF) [file pone.0054778.s008.tif]
